# Resting-state network alterations in depression: a comprehensive meta-analysis of functional connectivity

**DOI:** 10.1017/S0033291725000303

**Published:** 2025-02-26

**Authors:** Zhihui Zhang, Yijing Zhang, He Wang, Minghuan Lei, Yifan Jiang, Di Xiong, Yayuan Chen, Yujie Zhang, Guoshu Zhao, Yao Wang, Wanwan Zhang, Jinglei Xu, Ying Zhai, Qi An, Shen Li, Xiaoke Hao, Feng Liu

**Affiliations:** 1 Department of Radiology and Tianjin Key Laboratory of Functional Imaging & Tianjin Institute of Radiology, Tianjin Medical University General Hospital, Tianjin, China; 2 School of Nursing, Tianjin Medical University, Tianjin, China; 3 Department of Mathematics, Shanghai University, Shanghai, China; 4 Institute of Mental Health, Tianjin Anding Hospital, Mental Health Center of Tianjin Medical University, Tianjin, China; 5 Brain Assessment & Intervention Laboratory, Tianjin Anding Hospital, Mental Health Center of Tianjin Medical University, Tianjin, China; 6 School of Artificial Intelligence, Hebei University of Technology, Tianjin, China

**Keywords:** depression, fMRI, functional connectivity, neuroimaging meta-analysis, resting-state functional networks

## Abstract

**Background:**

Depression has been linked to disruptions in resting-state networks (RSNs). However, inconsistent findings on RSN disruptions, with variations in reported connectivity within and between RSNs, complicate the understanding of the neurobiological mechanisms underlying depression.

**Methods:**

A systematic literature search of PubMed and Web of Science identified studies that employed resting-state functional magnetic resonance imaging (fMRI) to explore RSN changes in depression. Studies using seed-based functional connectivity analysis or independent component analysis were included, and coordinate-based meta-analyses were performed to evaluate alterations in RSN connectivity both within and between networks.

**Results:**

A total of 58 studies were included, comprising 2321 patients with depression and 2197 healthy controls. The meta-analysis revealed significant alterations in RSN connectivity, both within and between networks, in patients with depression compared with healthy controls. Specifically, within-network changes included both increased and decreased connectivity in the default mode network (DMN) and increased connectivity in the frontoparietal network (FPN). Between-network findings showed increased DMN–FPN and limbic network (LN)–DMN connectivity, decreased DMN–somatomotor network and LN–FPN connectivity, and varied ventral attention network (VAN)–dorsal attentional network (DAN) connectivity. Additionally, a positive correlation was found between illness duration and increased connectivity between the VAN and DAN.

**Conclusions:**

These findings not only provide a comprehensive characterization of RSN disruptions in depression but also enhance our understanding of the neurobiological mechanisms underlying depression.

## Introduction

Depression is a global public health challenge that affects millions of people worldwide (Q. Liu et al., 2020a). This widespread mental health disorder impacts daily functioning and significantly contributes to the global burden of disability (Herrman et al., 2022). It manifests through persistent sadness, loss of interest or pleasure, feelings of guilt or low self-worth, and disturbances in sleep or appetite (Belmaker & Agam, 2008; Otte et al., 2016; Zahn et al., 2015). These symptoms can become chronic or recurrent, substantially impairing an individual’s ability to manage everyday activities (Richards, 2011). Despite these clear clinical presentations, the underlying biological mechanisms of depression are complex and not fully understood.

Resting-state networks (RSNs), identified through resting-state functional magnetic resonance imaging (fMRI), provide crucial insights into the biological basis of depression (Brakowski et al., 2017). These RSNs are established based on functional connectivity (FC), which refers to the temporal correlation of neural activity between different brain regions during a resting state (van den Heuvel & Hulshoff Pol, 2010). These networks, which consist of interconnected brain regions, play important roles in cognitive and emotional processes (Rolls, 2015; Smallwood et al., 2021). Two predominant methods for analyzing RSNs are seed-based analysis and independent component analysis (ICA) (Greicius, 2008). Seed-based analysis focuses on predefined brain regions, examining the correlations of their activity with other brain areas (Biswal, Yetkin, Haughton, & Hyde, 1995). In contrast, ICA identifies consistent patterns of brain activity across different regions in a data-driven manner (Allen, Erhardt, Wei, Eichele, & Calhoun, 2012). These techniques have uncovered disruptions in RSNs associated with the core symptoms of depression, such as pervasive sadness and anhedonia (Pan et al., 2022). However, research findings have often been inconsistent, involving both within-RSN connectivity, which refers to functional connections among regions within the same RSN, and between-RSN connectivity, which describes functional connections between regions belonging to different RSNs. For instance, some studies have reported increased connectivity within the default mode network (DMN) (Alexopoulos et al., 2012), which is associated with self-referential thoughts and emotional regulation (Sheline et al., 2009; Smallwood et al., 2021), while others have found reduced connectivity (Yan et al., 2019). Similar discrepancies arise in the limbic network (LN) (Lui et al., 2011; Pannekoek et al., 2014), which is essential for detecting and responding to emotion and reward-related decision-making (Rolls, 2015). Disparities also exist in the connectivity between networks, with conflicting reports on the relationship between the DMN and the frontoparietal network (FPN) (Bessette et al., 2018; Fettes et al., 2018). These inconsistencies likely stem from methodological differences, limited sample sizes, and variability in the demographics of the clinical populations.

Neuroimaging meta-analysis has emerged as a promising tool for synthesizing diverse findings across different studies, thereby resolving inconsistencies in depression research (Gray, Müller, Eickhoff, & Fox, 2020). A pioneering study published in 2015 utilized the multi-kernel density analysis (MKDA) method to conduct a meta-analysis investigating RSN dysfunctions in major depressive disorder (MDD), contributing to our understanding of this condition (Kaiser, Andrews-Hanna, Wager, & Pizzagalli, 2015). However, this study did not include ICA results, which could have provided additional insights into within-network alterations (Joel, Caffo, van Zijl, & Pekar, 2011). Additionally, the analysis included only a limited number of studies, whereas several recent publications on the role of RSNs in depression have used larger sample sizes (Y. Liu et al., 2020b; Zhou et al., 2022). Furthermore, the focus was solely on MDD, potentially limiting the generalizability of the findings across the spectrum of depressive disorders.

In the current study, we conducted a comprehensive neuroimaging meta-analysis to investigate RSN dysfunctions in depression using the anisotropic effect-size signed differential mapping (AES-SDM) method, which integrates voxel-based neuroimaging data by accounting for the spatial distribution of reported peak coordinates of differences (Radua & Mataix-Cols, 2009). Unlike MKDA, AES-SDM can combine both positive and negative coordinates in the same map, ensuring that the simultaneous presence of opposing effects at the same location can be accurately represented and analyzed. By including studies that used either seed-based or ICA methods, along with recently published studies, we sought to characterize consistent RSN disruptions in depression. Our findings contribute to the growing body of literature on the neural underpinnings of depression, offering a detailed understanding of RSN dysfunctions.

## Methods

### Literature search and selection

A systematic literature search was conducted on PubMed and Web of Science up to October 2023, targeting studies that employed resting-state fMRI to explore RSN changes in depression. The search terms included combinations of “depression” OR “depressive disorder” AND “FC” OR “functional connectivity” AND “resting” OR “rest” OR “functional magnetic resonance imaging” OR “neuroimaging” OR “fMRI.” Additionally, the reference lists of the included studies and relevant scholarly reviews were examined to identify further studies. For inclusion in our analysis, studies needed to meet several criteria: (1) they were peer-reviewed and published in English; (2) they compared groups of patients with depression to healthy controls (HCs); (3) they utilized resting-state fMRI with seed-based FC or ICA methodologies to examine RSNs; (4) their clinical diagnosis of depression was based on standardized diagnostic criteria, such as the Diagnostic and Statistical Manual of Mental Disorders (DSM); and (5) their results were reported in standard stereotaxic space (MNI or Talairach). Notably, node-based methods (e.g. graph theory) were not included in this meta-analysis, as these approaches primarily focus on global or node-specific network properties. While valuable in broader network analysis, node-based methods do not directly address within-network and between-network FC, which are the primary targets of our study. The exclusion criteria were as follows: (1) studies involving subjects with depression who also had other psychiatric disorders; (2) studies where the subject age range was not between 18 and 65 years; (3) studies that reported results only from region of interest (ROI)-to-ROI FC analysis; and (4) studies where between-group comparison coordinates could not be retrieved from original articles or through contact with authors. In cases of longitudinal designs, only the baseline comparison between patients and HCs was included. Our meta-analysis was conducted in accordance with the Preferred Reporting Items for Systematic Reviews and Meta-Analyses (PRISMA) guidelines (Page et al., 2021).

### Data extraction and coding

The following information was extracted from the analyzed studies: demographic information (e.g. sample size, age, and sex), clinical characteristics (e.g. illness duration and clinical scores), and resting-state fMRI data acquisition and processing parameters (e.g. types of MRI scanners, acquisition parameters, and smoothing kernel sizes). Coordinates for each seed were documented for the seed-based methods, and significant between-group comparison data, such as peak coordinates and statistical measures (e.g. *t*-values), were recorded for both the seed-based and ICA methods. In cases where studies did not report statistical values, “p” was assigned for positive peaks, and “n” was assigned for negative peaks.

The extracted coordinate data were coded as follows: all seed coordinates and the peak coordinates derived from between-group comparisons were converted to the MNI space (Lancaster et al., 2007). If the seed was a spherical ROI or an anatomical region from a standard anatomical atlas, the center of mass was calculated to obtain a representative coordinate. To assign seed and peak coordinates to the predefined RSNs, a 2 mm tolerance was applied. Specifically, a coordinate was categorized into an RSN if it was within 2 mm of any voxel in that RSN. If a coordinate fell within 2 mm of multiple RSNs, it was assigned to the RSN closest to the coordinate. This approach was carefully designed to address limitations in spatial resolution caused by fMRI preprocessing steps, such as spatial normalization and resampling. It also accommodated decimal coordinates derived from averaged ROIs (e.g. the center of mass) and accounted for minor anatomical variability across studies, ensuring a robust and consistent mapping to the predefined RSNs. Each coordinate was subsequently categorized into one of seven predefined RSNs established through whole-brain network parcellation, including the DMN, FPN, ventral attention network (VAN), dorsal attention network (DAN), visual network (VN), somatomotor network (SMN), and LN (Buckner, Krienen, Castellanos, Diaz, & Yeo, 2011; Choi, Yeo, & Buckner, 2012; Raut, Snyder, & Raichle, 2020; Yeo et al., 2011). The decision to use the Yeo 7-network parcellation, rather than the finer 17-network model (Yeo et al., 2011), was based on its balance between simplicity and biological interpretability. Based on the RSN assignment of seed and peak coordinates, studies were classified into within-network or between-network FC analyses. Specifically, a study was categorized as a within-network FC analysis if both the seed and its peak coordinate were within the same RSN. Conversely, it was categorized as a between-network FC analysis if the peak coordinate was located in a different RSN.

### Quality assessment

The quality and completeness of the included studies were evaluated using a 10-point checklist (Table S1), focusing on the demographic and clinical characteristics of the participants, the methods of image acquisition and analysis, and the robustness of the results and conclusions (Lin et al., 2023; Norman et al., 2016; Shepherd, Laurens, Matheson, Carr, & Green, 2012). Each criterion was scored as 0, 0.5, or 1, indicating whether it was not met, partially met, or fully met, respectively. The checklist results did not indicate overall study quality but rather the extent to which they met our study criteria.

### Meta-analysis

A voxel-wise meta-analysis was performed using AES-SDM software (version 5.15, https://www.sdmproject.com/) (Müller et al., 2018; Radua et al., 2012; Radua et al., 2014). For each RSN, two types of meta-analyses, within-network and between-network comparisons, were conducted if reported in at least 10 studies. The peak MNI coordinates and statistical values reflecting specific RSN alterations were entered into the AES-SDM software. Effect size and variance maps for the entire brain were reconstructed for each study using an anisotropic Gaussian kernel (full-width at half-maximum = 20 mm). These maps were then combined into a meta-analytic map using a random effects model that accounts for sample size, variability, and inter-study heterogeneity. The significance threshold was set at *p* < 0.001, with a peak height *Z* > 1 and a cluster size greater than 100 voxels to balance false positives and negatives (Zeng, Han, Gao, Sun, & Yuan, 2023).

### Sensitivity and exploratory analyses

To verify the reliability and reproducibility of our findings, we conducted sensitivity analyses. First, a jackknife sensitivity analysis was performed, repeating the voxel-wise meta-analysis each time with a different study excluded to determine whether the identified brain regions remained consistent across most combinations (Radua & Mataix-Cols, 2009). Second, between-study heterogeneity was assessed using Cochran’s *Q* statistic and the *I* ^2^ index to evaluate the consistency of results across studies (Higgins, Thompson, Deeks, & Altman, 2003). Third, potential publication bias for significant results was assessed using both funnel plots and Egger’s test, providing both visual and quantitative evaluations of bias (Ma et al., 2023).

To further explore connectivity patterns in networks excluded from the main analyses due to an insufficient number of studies (fewer than 10), exploratory analyses were performed using a lower threshold of 2 studies (Dong, Wang, Chang, Luo, & Yao, 2018; Kaiser et al., 2015). This approach allowed for the examination of within-network connectivity patterns in the LN (5 datasets), VN (4 datasets), VAN (3 datasets), DAN (2 datasets), and SMN (4 datasets). Between-network connectivity patterns involving the DAN (4 datasets) and SMN (2 datasets) as seed networks were also analyzed.

### Meta-Regression and subgroup analyses

Meta-regression analyses were conducted to explore the potential effects of illness duration and depression severity (i.e. Hamilton Depression Rating Scale) on between-group RSN differences. Specifically, the mean effect size of each significant cluster was extracted from each study, and linear regression analyses were performed across studies to correlate these effect sizes with study-level continuous variables. Given the exploratory nature of this analysis, the significance level was set at an uncorrected *p* < 0.05.

Subgroup analyses were also conducted to assess the potential influence of global signal regression (GSR) and MRI scanner type on FC patterns. For the GSR analysis, studies were divided into those that applied GSR and those that did not, while for the scanner type analysis, studies were categorized based on the type of MRI scanner used (GE versus Siemens, Philips scanners were excluded due to insufficient data for analysis). Due to an insufficient number of datasets, additional subgroup analyses could not be performed. Considering the limited statistical power of these analyses, only uncorrected results (i.e. unthresholded statistical maps) are presented to minimize the risk of overinterpretation.

## Results

### Overview of the included studies

**Figure 1.**
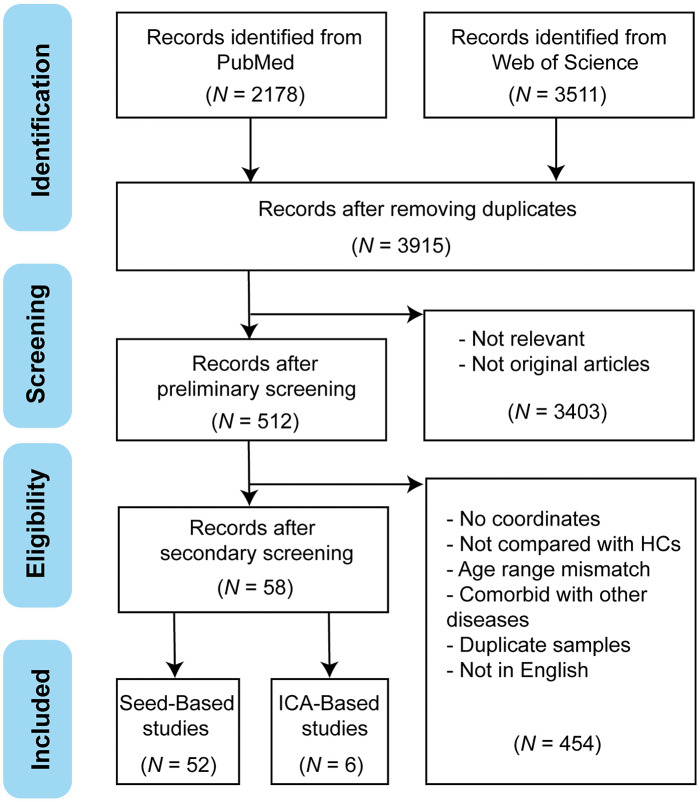
The flowchart of the search strategy and retrieved studies according to the PRISMA guidelines. Abbreviations: HCs, healthy controls; ICA, independent component analysis; N, number; PRISMA, preferred reporting items for systematic reviews and meta-analysis.

Our systematic search and selection process, shown in Figure 1, initially identified 5689 records from PubMed and Web of Science. Following the removal of duplicates, 3915 unique studies were retained for further screening. After rigorous screening, 58 studies were included in our meta-analysis (supplementary results), and the quality assessment scores assigned to the included studies are shown in Table S2. All included studies received quality assessment scores greater than 9, reflecting their high quality across multiple dimensions, including participant evaluation and sample characteristics, imaging acquisition and statistical methods, as well as results reporting and interpretation. Among these studies, 52 employed a seed-based approach, and 6 used the ICA method, involving 2321 individuals with depression and 2197 HCs. Notably, the screening process revealed that two studies utilized the same sample but were treated as distinct studies due to the selection of different seeds (Guo, Liu, Liu, et al., 2015a; Guo, Liu, Xiao, et al., 2015b). The demographic, clinical, and statistical information is summarized in Tables S3–S5. Two-sample *t*-tests revealed no significant differences in age (*p* = 0.645) or sex distribution (female-to-male ratio, *p* = 0.145) between the patient and control groups.

The studies included in this meta-analysis were categorized into seven predefined RSNs based on their seed and peak coordinates. Specifically, our meta-analysis included 24 studies on the DMN and 13 on the FPN for within-network analysis. For between-network analysis, 24 studies examined the DMN seeds, 14 focused on the LN seeds, and 11 investigated the VAN seeds. Due to insufficient data (fewer than 10 studies), other seed networks were not included in this meta-analysis. Further details about seed categorization are provided in Tables S6–S7.

### Within-network and between-network FC changes

**Figure 2.**
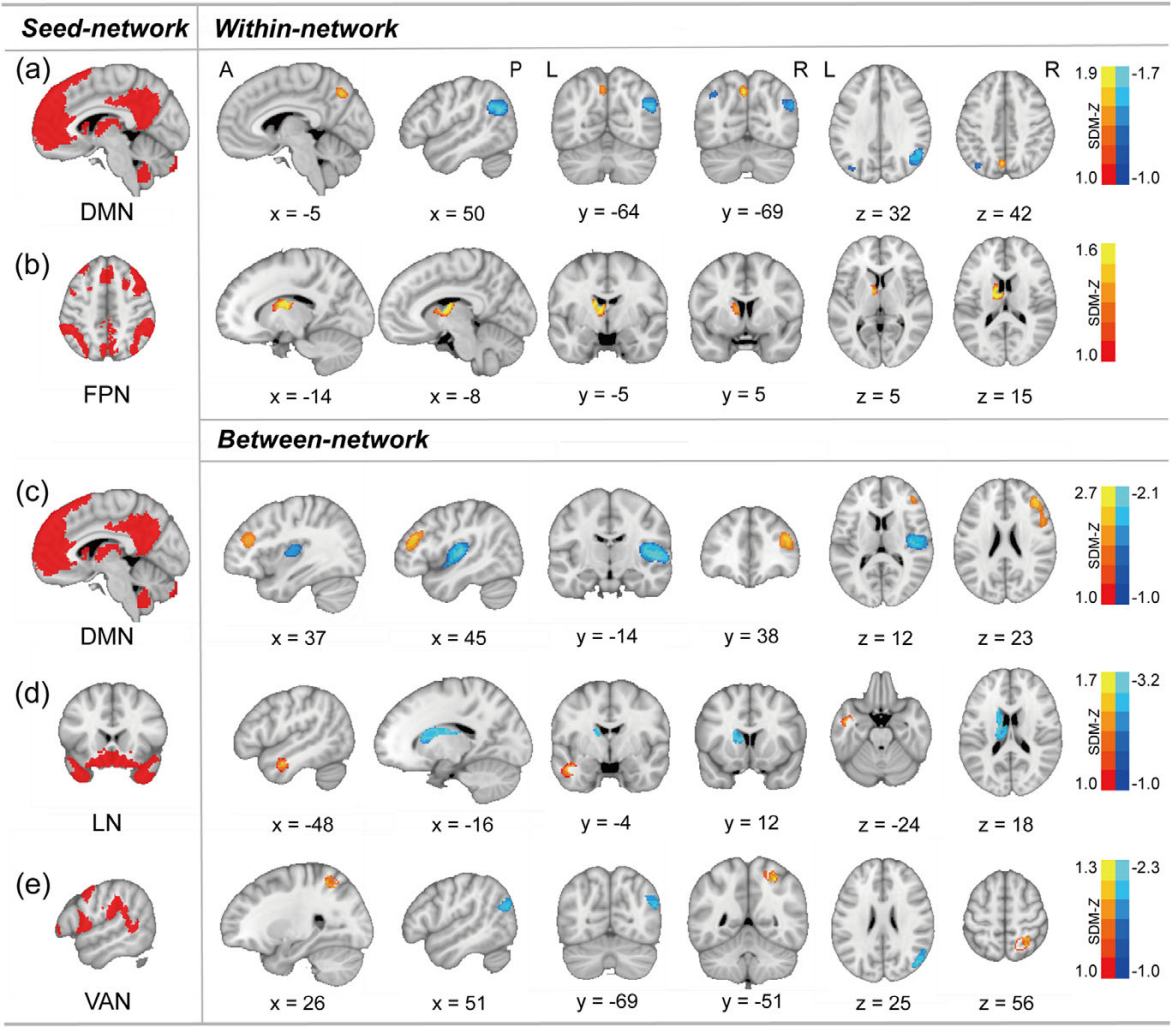
Meta-analysis results of significant RSN changes in depression. The results are presented as follows: (a) within the DMN, (b) within the FPN, (c) between the DMN and other networks, (d) between the LN and other networks, and (e) between the VAN and other networks. The colorbar represents SDM-*Z* values, with warm and cold colors indicating increased and decreased functional connectivity in patients with depression, respectively. Abbreviations: DAN, dorsal attention network; DMN, default mode network; FPN, frontoparietal network; HCs, healthy controls; LN, limbic network; RSN, resting-state network; SDM, seed-based *d* mapping; SMN, somatosensory network; VAN, ventral attention network.

In individuals with depression, FC changes within the DMN were variable, with both increases and decreases observed compared with HCs. Increased connectivity was noted between DMN seeds and the left precuneus, while decreased connectivity was found between DMN seeds and regions such as the bilateral angular gyrus and the right middle temporal gyrus (Table 1, Figure 2a, Figure S1a). The FPN exhibited increased connectivity between its seeds and the left caudate nucleus (Table 1, Figure 2b, Figure S1b).

**Table 1.** Summary of the results of the meta-analysis, heterogeneity test, and Egger’s test

Seed-network	Target-network	Target-anatomy	Peak MNI coordinates			SDM-*Z*	*p*-value	Cluster size	Heterogeneity test		Egger’s test
			X	Y	Z			voxels	*Q* (*p*-value)	*I* ^2^ (%)	*p*-value
Within-network											
DMN	DMN	L-PCU	–4	−68	44	1.64	2.58E–05	123	16.25 (0.84)	0.03	0.74
DMN	DMN	R-ANG/R-MTG	48	−60	28	−1.87	~0	667	19.58 (0.67)	3.57	0.96
DMN	DMN	L-ANG	−38	−72	38	−1.39	5.88E–04	133	29.88 (0.15)	21.46	0.48
FPN	FPN	L-CAU	−6	−2	10	1.57	5.19E–06	325	10.12 (0.61)	4.39	0.65
Between-network											
DMN	FPN	R-MFG/R-IFG	44	34	24	2.34	2.58E–05	705	17.26 (0.80)	1.60	0.84
DMN	SMN	R-STG/R-HES	44	−16	8	−2.08	~0	1275	17.38 (0.79)	0.33	0.83
LN	DMN	L-MTG/L-ITG	−46	−2	−24	1.55	2.06E–05	186	8.97 (0.78)	0.85	0.46
LN	FPN	L-CAU	−14	4	22	−3.17	~0	473	12.19 (0.51)	5.14	0.06
VAN	DAN	R-IPL/R-SPL	30	−50	54	1.32	1.29E–04	239	11.78 (0.30)	14.24	0.89
VAN	DAN	R-MOG	46	−74	28	−2.22	1.03E–05	355	10.50 (0.40)	10.83	0.90

Note: The positive and negative SDM-*Z* values represent the increase and decrease in functional connectivity in patients with depression, respectively.

Abbreviations: DAN, dorsal attention network; DMN, default mode network; FPN, frontoparietal network; L-ANG, left angular gyrus; L-CAU, left caudate nucleus; L-ITG, left inferior temporal gyrus; L-MTG, left middle temporal gyrus; LN, limbic network; L-PCU, left precuneus; MNI, Montreal Neurological Institute; *Q*, Cochran’s *Q* statistic; R-ANG, right angular gyrus; R-HES, right Heschl’s gyrus; R-IFG, right inferior frontal gyrus; R-IPL, right inferior parietal lobule; R-MFG, right middle frontal gyrus; R-MOG, right middle occipital gyrus; R-MTG, right middle temporal gyrus; R-SPL, right superior parietal lobule; R-STG, right superior temporal gyrus; SDM, seed-based *d* mapping; SMN, somatosensory network; VAN, ventral attention network.

Depression was also associated with altered between-network FC. Increased connectivity was observed between DMN seeds and FPN regions, including the right middle frontal gyrus and right inferior frontal gyrus. In contrast, decreased connectivity occurred between DMN seeds and SMN regions, specifically the right superior temporal gyrus and the right Heschl’s gyrus (Table 1, Figure 2c, Figure S1c). Connectivity between LN seeds and DMN regions, such as the left middle temporal gyrus and left inferior temporal gyrus, was heightened, while the connectivity between LN seeds and FPN regions, including the left caudate nucleus, was reduced (Table 1, Figure 2d, Figure S1d). Additionally, variable connectivity patterns were observed between the VAN seeds and DAN regions: heightened connectivity with the right inferior and superior parietal lobules, and reduced connectivity with the right middle occipital gyrus (Table 1, Figure 2e, Figure S1e).

**Figure 3.**
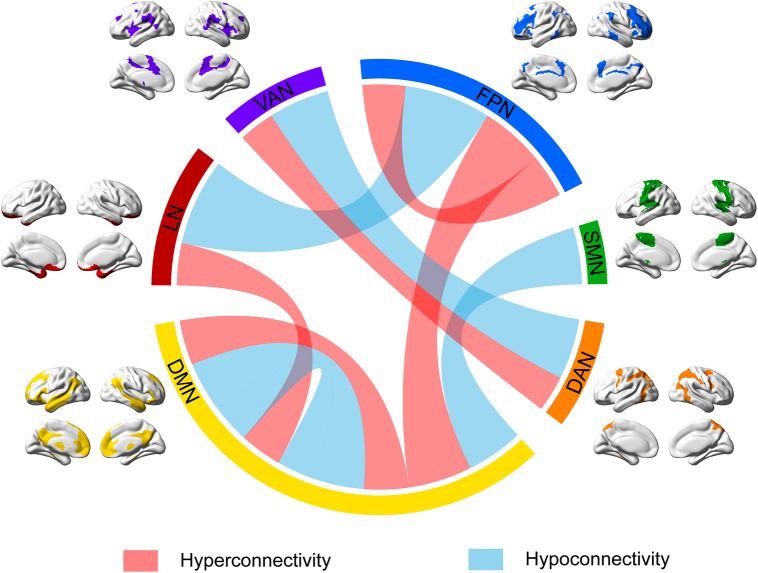
Comprehensive map of RSN alterations in depression. The curves depict alterations in functional connectivity both within and between RSNs, while the outer ring colors are used solely to distinguish different RSNs. Increased connectivity (hyperconnectivity) is represented by red curves, while decreased connectivity (hypoconnectivity) is represented by blue curves. Within the DMN, both increased and decreased connectivity were observed. The FPN showed an overall increase in connectivity across its regions. Between networks, increased connectivity was found between the DMN and FPN, while decreased connectivity was observed between the DMN and SMN. Additionally, enhanced connectivity was observed between the LN and DMN, while decreased connectivity was found between the LN and FPN. Both increased and decreased connectivity were observed between the VAN and DAN. Abbreviations: DAN, dorsal attention network; DMN, default mode network; FPN, frontoparietal network; LN, limbic network; RSN, resting-state network; SMN, somatosensory network; VAN, ventral attention network.

A summary of these alterations across multiple RSNs, highlighting patterns of hyperconnectivity and hypoconnectivity in depression, is visualized in Figure 3.

### Sensitivity and exploratory analyses

**Figure 4.**
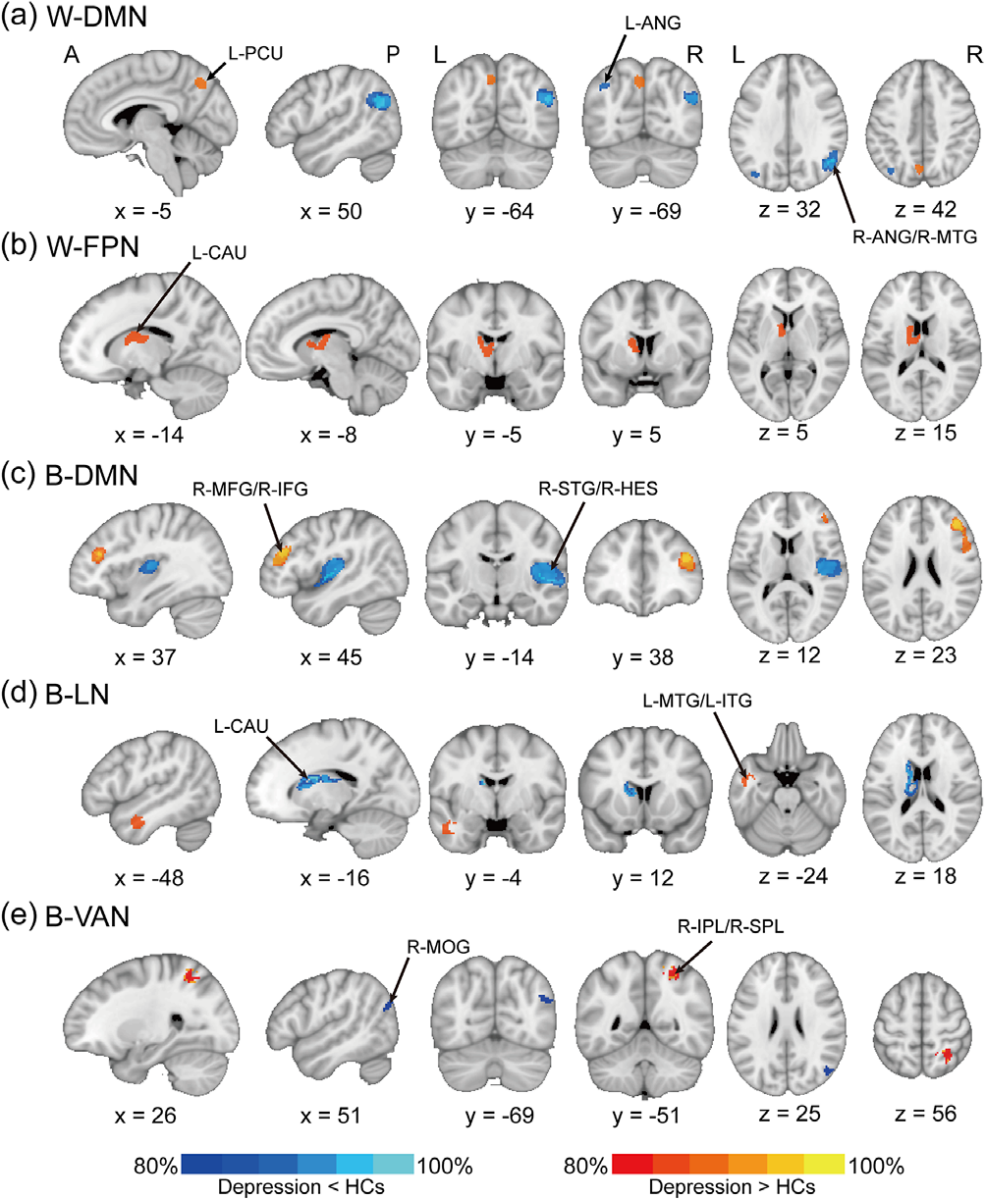
Results of the jackknife sensitivity analysis. Regions surviving more than 80% of iterations are shown for: (a) within-DMN, (b) within-FPN, (c) between DMN and other networks, (d) between LN and other networks, and (e) between VAN and other networks. The colorbar represents reproducibility rates, with warm and cool colors indicating regions of increased and decreased functional connectivity, respectively. Abbreviations: B, between; DMN, default mode network; FPN, frontoparietal network; HCs, healthy controls; L-ANG, left angular gyrus; L-CAU, left caudate nucleus; L-ITG, left inferior temporal gyrus; L-MTG, left middle temporal gyrus; LN, limbic network; L-PCU, left precuneus; R-ANG, right angular gyrus; R-HES, right Heschl’s gyrus; R-IFG, right inferior frontal gyrus; R-IPL, right inferior parietal lobule; R-MFG, right middle frontal gyrus; R-MOG, right middle occipital gyrus; R-MTG, right middle temporal gyrus; R-SPL, right superior parietal lobule; R-STG, right superior temporal gyrus; VAN, ventral attention network; W, within.

Jackknife sensitivity analyses confirmed the robustness of the findings, with consistent results in the left caudate nucleus and right middle frontal gyrus across all study combinations, while other clusters remained stable in over 80% of the iterations (Figure 4). No significant heterogeneity or publication bias was detected, as indicated by Cochran’s *Q* statistic, the *I* ^2^ index, Egger’s test results, or funnel plot results (Table 1, Figure S2). Exploratory analyses using a lower threshold of 2 studies did not identify significant within-network connectivity in the LN, VN, VAN, DAN, or SMN, nor significant between-network connectivity involving the DAN or SMN as seed networks.

### Meta-regression and subgroup analyses

Meta-regression analyses revealed a significant positive correlation between illness duration and increased FC between the VAN and DAN (*p* = 0.0013, *t* = 3.2204). No significant associations were observed between depression severity (HAMD scores) and FC changes across other RSNs or clusters. The detailed meta-regression results are provided in Table S8.

The subgroup analyses demonstrated both overlaps and differences in RSN findings across studies based on factors such as the application of GSR and the type of MRI scanner used (GE versus Siemens). While shared regions were observed across subgroups, substantial variations highlight the influence of methodological and technical factors on the detected connectivity alterations (Figures S3-S4). These findings reflect the complexity of RSN connectivity changes in depression and the impact of methodological variability.

## Discussion

In this study, we conducted a comprehensive neuroimaging meta-analysis to investigate RSN dysfunctions in depression. By including 58 studies that utilized both seed-based and ICA methods, along with recent publications, our meta-analysis analyzed data from 2321 individuals with depression and 2197 HCs. The results revealed a complex pattern of FC changes: mixed alterations within the DMN, increased connectivity within the FPN, and varied connectivity between key networks. Specifically, we observed altered connectivity between the DMN and FPN, the DMN and SMN, the LN and DMN, the LN and FPN, and the VAN and DAN. These results highlight alterations in RSNs associated with depression, providing valuable insights into the underlying neurobiological mechanisms involved.

Our analysis identified varying FC within the DMN in individuals with depression. The increased connectivity observed between the DMN and the left precuneus is consistent with heightened self-referential processing (Broyd et al., 2009; Dadario & Sughrue, 2023; X. Liu et al., 2024), which is a core feature of depressive rumination (Nejad, Fossati, & Lemogne, 2013). The observed heightened connectivity could indicate an overactive internal thought process, contributing to the persistent negative thoughts characteristic of depression (LeMoult & Gotlib, 2019). Conversely, decreased connectivity in the DMN with regions such as the bilateral angular gyrus and right middle temporal gyrus may indicate disrupted integration of external information and impaired cognitive functioning (Mo et al., 2020). These results align with previous studies suggesting both hyperactivity and hypoactivity within the DMN in depression (Broyd et al., 2009; Yan et al., 2019), reflecting a balance between excessive internal focus and diminished external engagement (Nolen-Hoeksema, Wisco, & Lyubomirsky, 2008; Watson, Harvey, McCabe, & Reynolds, 2020). The dual nature of these findings highlights the complexity of DMN alterations and their role in the cognitive and emotional disturbances seen in depression. Furthermore, the FPN showed increased within-network connectivity between its seeds and the left caudate nucleus, a region implicated in reward processing and cognitive control (Pizzagalli et al., 2009). This enhancement may represent compensatory mechanisms aimed at maintaining cognitive functions despite depressive symptoms (Schultz et al., 2019). Increased connectivity within the FPN could be an adaptive response to counteract the cognitive deficits associated with depression, suggesting that the brain attempts to preserve functionality in key cognitive areas.

Our between-network analysis highlighted increased connectivity between the DMN seeds and FPN regions, such as the right middle frontal gyrus and the right inferior frontal gyrus. This finding suggested enhanced communication between networks involved in self-referential thought and executive control (Dixon et al., 2018; Sheline et al., 2009), potentially contributing to the persistent negative thoughts and cognitive inflexibility characteristic of depression (LeMoult & Gotlib, 2019). Enhanced DMN–FPN connectivity may exacerbate the cycle of rumination and difficulty in shifting attention away from negative stimuli, a core feature of depressive cognition. In contrast, decreased connectivity between DMN seeds and SMN regions (right superior temporal gyrus and right Heschl’s gyrus) indicates disrupted sensorimotor integration, which could manifest as psychomotor retardation (Yang et al., 2021), a common symptom in depression. This reduced connectivity could impair the coordination between thought and movement, leading to the slowed physical and cognitive responses typical of depressed individuals.

Increased connectivity between LN seeds and DMN regions, along with decreased connectivity between LN seeds and FPN regions, reflects a complex interplay between emotional processing and cognitive control systems (Marchetti, Koster, Sonuga-Barke, & De Raedt, 2012; Sequeira et al., 2007), potentially underpinning emotional dysregulation and executive dysfunction in depression (Dixon et al., 2018; Sequeira et al., 2007). This pattern suggests that while emotional and self-referential processing is heightened, the ability to regulate these processes through cognitive control is diminished. Moreover, enhanced connectivity between the VAN seeds and DAN regions, coupled with reduced connectivity between other VAN seeds and DAN areas, reveals a distinct alteration in attentional networks (Vossel, Geng, & Fink, 2014). The observed patterns suggest both hyperactivity and hypoactivity within attentional systems, possibly contributing to the attentional biases and difficulties in focusing commonly reported by depressed individuals (J. Liu et al., 2019; Sylvester et al., 2013). These alterations in attentional networks could underlie the pervasive difficulty in concentrating and the susceptibility to distraction seen in depression. Additionally, meta-regression analyses revealed a positive correlation between illness duration and increased VAN–DAN connectivity, suggesting that longer periods of depression are linked to more pronounced abnormalities in the connectivity between these attentional networks. This heightened connectivity may signify either compensatory mechanisms or maladaptive neural plasticity in response to prolonged depressive symptoms, such as cognitive biases and attentional deficits, with sustained activation potentially leading to over-recruitment of attentional resources and further amplifying these changes. Consequently, VAN–DAN connectivity holds promise as a biomarker for the chronicity of depression, offering the potential for early identification of high-risk individuals and guiding the development of targeted therapeutic strategies.

Our study’s results were compared with those of Kaiser et al. (2015), who used the MKDA method for a meta-analysis on network dysfunctions in MDD. Similar to Kaiser et al., we observed altered connectivity within the DMN and FPN. However, our study uniquely identified increased connectivity between the DMN and SMN, between the LN and FPN, and between the VAN and DAN. In contrast, we did not replicate Kaiser et al.’s findings of altered connectivity between the FPN and DMN, or between the LN and DMN. These discrepancies can be attributed to several factors. First, our inclusion of several newly published RSN-related studies and the integration of ICA studies could enhance the statistical power to detect new results. Second, AES-SDM offers advantages over MKDA by combining both positive and negative coordinates in the same map, which provides a more accurate and comprehensive representation of the RSN dysfunctions involved in depression (Radua & Mataix-Cols, 2009). Finally, our meta-analysis included studies on depression diagnosed using DSM or ICD criteria without being restricted to MDD, whereas Kaiser et al. focused exclusively on MDD (Kaiser et al., 2015). By adopting broader inclusion criteria, our study encompasses a wider spectrum of clinically diagnosed depressive conditions, including but not limited to MDD. This approach enables the identification of RSN alterations that are shared across various forms of depression and provides insights into the neurobiological underpinnings that may contribute to a more generalized understanding of depression.

Our meta-analysis also highlights the complexities and challenges associated with integrating findings from heterogeneous datasets in resting-state fMRI research. While our primary goal was to identify consistent patterns of FC alterations in depression, the subgroup analyses revealed both consistencies and inconsistencies in the findings, particularly when datasets were stratified by factors such as the application of GSR and differences in scanner manufacturers (GE versus Siemens). Specifically, overlapping findings were observed in several brain regions across subgroups, indicating robust alterations in networks such as the DMN and VAN. However, substantial differences emerged in certain regions, underscoring the influence of preprocessing choices and technical variability on the observed results. For example, GSR—a widely debated preprocessing step (Murphy & Fox, 2017)—appears to differentially impact the detection of FC alterations, as demonstrated by the distinct patterns observed between the GSR-Y and GSR-N groups (Figure S3). Similarly, scanner-specific variability contributed to differences in regional FC changes, as shown in the subgroup analysis comparing the GE and Siemens datasets (Figure S4). These inconsistencies highlight the dual role of methodological variability: while it introduces challenges in interpreting results, it also offers valuable insights into how technical and analytical factors shape findings in depression-related FC studies. Importantly, the small number of datasets within certain subgroups (e.g. GSR-Y) limits the generalizability of these findings, emphasizing the need for larger, harmonized datasets in future research. To address these challenges, future studies should prioritize the standardization of preprocessing pipelines and adopt advanced harmonization methods, such as the ComBat approach (Johnson, Li, & Rabinovic, 2007; Yu et al., 2018), to mitigate scanner and preprocessing effects. The observed inconsistent patterns in key networks across subgroups underscore the importance of considering methodological variability when interpreting meta-analytic findings and highlight the potential for further refinement in this field.

In this study, we applied a threshold of at least 10 studies for conducting within-network and between-network analyses, along with a criterion requiring a sample size of >10 participants per group in our quality assessment (see Table S1 for details). These thresholds were chosen to ensure robust and reliable findings while balancing statistical power and data availability (Müller et al., 2018). Although no universal guidelines exist for the minimum number of studies required in neuroimaging meta-analyses, thresholds as low as 2 studies have been used in prior research (Dong et al., 2018; Kaiser et al., 2015). However, a larger number of studies enhances statistical power and reduces the impact of variability across individual studies. Exploratory analyses using a lower threshold of 2 studies did not yield other significant results, likely due to the small datasets and inconsistencies across studies, further validating the robustness of our chosen threshold of 10. Similarly, the sample size >10 criterion aligns with established neuroimaging standards, as smaller sample sizes are more prone to variability and reduced statistical power. Power simulations have demonstrated that detecting moderate effects in fMRI studies with 80% power and an alpha level of 0.05 typically requires a total sample size of 11–12 participants; however, for smaller effects, the required sample size increases, with over 20 participants needed to maintain 80% power (Desmond & Glover, 2002). Although this criterion was not derived from specific power calculations for individual studies included in the meta-analysis, it reflects an effort to ensure the reliability of the included studies while maintaining consistency with prior quality assessment frameworks. By explicitly addressing these thresholds, our study highlights the trade-offs in meta-analytic design and contributes to ongoing discussions about best practices in neuroimaging meta-analyses.

This study has several limitations that should be acknowledged. First, our focus on a limited number of RSNs due to the number of available studies may have led to overlooking potential changes in other networks. Second, relying on published peak coordinates restricts our ability to capture a comprehensive view of RSN changes. Third, the lack of key experimental details, such as confirmation of full-brain coverage during image acquisition in many included studies (Table S4), limits our ability to fully assess study quality. Fourth, head motion is an inherent limitation in resting-state FC studies. While all included studies implemented standard preprocessing steps, such as realignment, motion parameter regression, and ICA to remove motion-related components, residual effects cannot be entirely eliminated. Fifth, the use of GSR varied across the included studies. Subgroup analyses were performed to examine the potential influence of GSR on the findings, and we provided both regression and non-regression subgroup results for comparison. However, the lack of consensus regarding the use of GSR in resting-state fMRI research (Murphy & Fox, 2017) and the mixed results observed in our subgroup analyses suggest that this remains an open question for future investigations. Finally, the interpretation of the directionality and functional significance of FC changes presents a significant challenge. While our interpretations were informed by established functional roles of specific networks, these interpretations are inherently context-dependent and may vary across individuals, symptom profiles, or illness stages. The absence of individual-level clinical or behavioral data in this meta-analysis further limits our ability to directly link FC alterations to specific depressive symptoms or outcomes. Future longitudinal studies incorporating multimodal imaging and detailed clinical assessments are necessary to explore these relationships further and refine the understanding of FC changes in depression.

In conclusion, our meta-analysis identified significant disruptions in RSNs associated with depression. Specifically, we identified increased FC within the DMN and FPN, as well as between the DMN and FPN, indicating heightened self-referential and executive control processes. Conversely, decreased FC between the DMN and SMN suggests impaired sensory and motor integration. Additionally, enhanced connectivity was found between the LN and DMN, and between LN and DMN, while connectivity decreased between the LN and FPN. Both hypoconnectivity and hyperconnectivity were observed between the VAN and DAN, and between DMN and SMN. These consistent patterns provide critical insights into the neurobiological underpinnings of depression, emphasizing the importance of RSN-focused research in understanding and addressing this disorder.

## Supporting information

Zhang et al. supplementary material Zhang et al. supplementary material

## Supplementary material

To view supplementary material for this article, please visit http://doi.org/10.1017/S0033291725000303.
