# Supplementary material for: Resting-state network alterations in depression: a comprehensive meta-analysis of functional connectivity
Source: Psychol Med. 2025 Feb 26;55:e63. doi: 10.1017/S0033291725000303 (PMC12080655; doi:10.1017/S0033291725000303)
Supplement: Zhang et al. supplementary material [file S0033291725000303sup001.docx]

**Online Supplementary Material**

**Resting-state Network Alterations in Depression: A Comprehensive Meta-analysis of Functional Connectivity**

Zhihui Zhang^1#^, Yijing Zhang^1#^, He Wang^1#^, Minghuan Lei^1^, Yifan Jiang^2^, Di Xiong^3^, Yayuan Chen^1^, Yujie Zhang^1^, Guoshu Zhao^1^, Yao Wang^1^, Wanwan Zhang^1^, Jinglei Xu^1^, Ying Zhai^1^, Qi An^1^, Shen Li^4,5^*, Xiaoke Hao^6^*, Feng Liu^1^*

**Affiliations:**

^1^Department of Radiology and Tianjin Key Laboratory of Functional Imaging & Tianjin Institute of Radiology, Tianjin Medical University General Hospital, Tianjin 300052, China

^2^School of Nursing, Tianjin Medical University, Tianjin 300070, China

^3^Department of Mathematics, Shanghai University, Shanghai 200444, China

^4^Institute of Mental Health, Tianjin Anding Hospital, Mental Health Center of Tianjin Medical University, Tianjin 300222, China

^5^Brain Assessment & Intervention Laboratory, Tianjin Anding Hospital, Mental Health Center of Tianjin Medical University, Tianjin 300222, China

^6^School of Artificial Intelligence, Hebei University of Technology, Tianjin 300401, China

^#^These authors contributed equally to this study

***Correspondence to:**

Feng Liu, Department of Radiology, Tianjin Medical University General Hospital, No. 154, Anshan Road, Heping District, Tianjin 300052, China.

E-mail: fengliu@tmu.edu.cn

Xiaoke Hao, School of Artificial Intelligence, Hebei University of Technology, No. 5340, Xiping Road, Beichen District, Tianjin 300401, China.

E-mail: haoxiaoke@hebut.edu.cn

Shen Li, Institute of Mental Health, Tianjin Anding Hospital, Mental Health Center of Tianjin Medical University, No. 13, Liulin Road, Hexi District, Tianjin 300222, China.

E-mail: lishen@tmu.edu.cn

**Contents**

Supplementary Results 4

The 58 studies included in this meta-analysis 4

Seed-based analysis (52 studies) 4

ICA studies (6 studies) 14

Supplementary Tables 16

Table S1. The 10-point checklist of quality assessment for included studies. 16

Table S2. Quality assessment scores of included studies. 17

Table S3. The demographic and clinical information of included studies. 19

Table S4. The MRI acquisition and preprocessing details of included studies. 22

Table S5. The statistical overview of included studies. 27

Table S6. Summary of seed-networks and anatomical regions of included studies in this meta-analysis. 37

Table S7. Summary of information included in ICA studies in this meta-analysis. 41

Table S8. Meta-regression analysis: associations of RSN alterations with illness duration and HAMD scores in depression. 42

Supplementary Figures 43

Figure S1. Meta-analysis results of significant RSN changes in depression (unthresholded). 43

Figure S2. Funnel plots of regions with significantly altered functional connectivity. 44

Figure S3. Subgroup analysis of the effect of GSR on functional connectivity in depression. 45

Figure S4. Subgroup analysis of the impact of MRI scanner type (GE vs. Siemens) on functional connectivity in depression. 46

References for Supplementary Materials 47

Supplementary Results

The 58 studies included in this meta-analysis

Seed-based analysis (52 studies)

Beckmann, F.-E., Seidenbecher, S., Metzger, C. D., Gescher, D. M., Carballedo, A., Tozzi, L., . . . Frodl, T. (2022). C-reactive protein is related to a distinct set of alterations in resting-state functional connectivity contributing to a differential pathophysiology of major depressive disorder. *Psychiatry Res Neuroimaging, 321*, 111440. doi:10.1016/j.pscychresns.2022.111440

Bessette, K. L., Jenkins, L. M., Skerrett, K. A., Gowins, J. R., DelDonno, S. R., Zubieta, J. K., . . . Langenecker, S. A. (2018). Reliability, Convergent Validity and Time Invariance of Default Mode Network Deviations in Early Adult Major Depressive Disorder. *Front Psychiatry, 9*, 244. doi:10.3389/fpsyt.2018.00244

Bluhm, R., Williamson, P., Lanius, R., Théberge, J., Densmore, M., Bartha, R., . . . Osuch, E. (2009). Resting state default-mode network connectivity in early depression using a seed region-of-interest analysis: decreased connectivity with caudate nucleus. *Psychiatry Clin Neurosci, 63*(6), 754-761. doi:10.1111/j.1440-1819.2009.02030.x

Cao, X., Liu, Z., Xu, C., Li, J., Gao, Q., Sun, N., . . . Zhang, K. (2012). Disrupted resting-state functional connectivity of the hippocampus in medication-naive patients with major depressive disorder. *J Affect Disord, 141*(2-3), 194-203. doi:10.1016/j.jad.2012.03.002

Chen, C., Liu, Z., Zuo, J., Xi, C., Long, Y., Li, M. D., . . . Yang, J. (2021). Decreased cortical folding of the fusiform gyrus and its hypoconnectivity with sensorimotor areas in major depressive disorder. *J Affect Disord, 295*, 657-664. doi:10.1016/j.jad.2021.08.148

Chen, F.-j., Gu, C.-z., Zhai, N., Duan, H.-f., Zhai, A.-l., & Zhang, X. (2020). Repetitive transcranial magnetic stimulation improves amygdale functional connectivity in major depressive disorder. *Front Psychiatry, 11*, 732. doi:10.3389/fpsyt.2020.00732

Chen, Y., Wang, C., Zhu, X., Tan, Y., & Zhong, Y. (2015). Aberrant connectivity within the default mode network in first-episode, treatment-naive major depressive disorder. *J Affect Disord, 183*, 49-56. doi:10.1016/j.jad.2015.04.052

Crowther, A., Smoski, M. J., Minkel, J., Moore, T., Gibbs, D., Petty, C., . . . Dichter, G. S. (2015). Resting-state connectivity predictors of response to psychotherapy in major depressive disorder. *Neuropsychopharmacology, 40*(7), 1659-1673. doi:10.1038/npp.2015.12

Cui, G., Wang, Y., Wang, X., Zheng, L., Li, L., Li, P., . . . Meng, X. (2020). Static and dynamic functional connectivity of the prefrontal cortex during resting-state predicts self-serving bias in depression. *Behav Brain Res, 379*, 112335. doi:10.1016/j.bbr.2019.112335

Davey, C. G., Harrison, B. J., Yücel, M., & Allen, N. (2012). Regionally specific alterations in functional connectivity of the anterior cingulate cortex in major depressive disorder. *Psychol Med, 42*(10), 2071-2081. doi:10.1017/S0033291712000323

de Kwaasteniet, B., Ruhe, E., Caan, M., Rive, M., Olabarriaga, S., Groefsema, M., . . . Denys, D. (2013). Relation between structural and functional connectivity in major depressive disorder. *Biol Psychiatry, 74*(1), 40-47. doi:10.1016/j.biopsych.2012.12.024

de Kwaasteniet, B. P., Rive, M. M., Ruhé, H. G., Schene, A. H., Veltman, D. J., Fellinger, L., . . . Denys, D. (2015). Decreased resting-state connectivity between neurocognitive networks in treatment resistant depression. *Front Psychiatry, 6*, 125963. doi:10.3389/fpsyt.2015.00028

Duan, G., He, Q., Pang, Y., Chen, W., Liao, H., Liu, H., . . . Zhang, J. (2020). Altered amygdala resting-state functional connectivity following acupuncture stimulation at BaiHui (GV20) in first-episode drug-Naïve major depressive disorder. *Brain Imaging Behav, 14*, 2269-2280. doi:10.1007/s11682-019-00178-5

Fettes, P. W., Moayedi, M., Dunlop, K., Mansouri, F., Vila-Rodriguez, F., Giacobbe, P., . . . Downar, J. (2018). Abnormal Functional Connectivity of Frontopolar Subregions in Treatment-Nonresponsive Major Depressive Disorder. *Biol Psychiatry Cogn Neurosci Neuroimaging, 3*(4), 337-347. doi:10.1016/j.bpsc.2017.12.003

Frodl, T., Bokde, A. L., Scheuerecker, J., Lisiecka, D., Schoepf, V., Hampel, H., . . . Meisenzahl, E. (2010). Functional connectivity bias of the orbitofrontal cortex in drug-free patients with major depression. *Biol Psychiatry, 67*(2), 161-167. doi:10.1016/j.biopsych.2009.08.022

Furman, D. J., Hamilton, J. P., & Gotlib, I. H. (2011). Frontostriatal functional connectivity in major depressive disorder. *Biol Mood Anxiety Disord, 1*, 1-11. doi:10.1186/2045-5380-1-11

Ge, R., Torres, I., Brown, J. J., Gregory, E., McLellan, E., Downar, J. H., . . . Vila-Rodriguez, F. (2019). Functional disconnectivity of the hippocampal network and neural correlates of memory impairment in treatment-resistant depression. *J Affect Disord, 253*, 248-256. doi:10.1016/j.jad.2019.04.096

Guo, W., Liu, F., Liu, J., Yu, M., Zhang, Z., Liu, G., . . . Zhao, J. (2015). Increased cerebellar-default-mode-network connectivity in drug-naive major depressive disorder at rest. *Medicine (Baltimore), 94*(9), e560. doi:10.1097/MD.0000000000000560

Guo, W., Liu, F., Xiao, C., Zhang, Z., Liu, J., Yu, M., . . . Zhao, J. (2015). Decreased insular connectivity in drug-naive major depressive disorder at rest. *J Affect Disord, 179*, 31-37. doi:10.1016/j.jad.2015.03.028

Hao, Z. Y., Zhong, Y., Ma, Z. J., Xu, H. Z., Kong, J. Y., Wu, Z., . . . Zhang, N. (2020). Abnormal resting-state functional connectivity of hippocampal subfields in patients with major depressive disorder. *BMC Psychiatry, 20*, 1-11. doi:10.1186/s12888-020-02490-7

He, Z., Lu, F., Sheng, W., Han, S., Long, Z., Chen, Y., . . . Ouyang, A. (2019). Functional dysconnectivity within the emotion-regulating system is associated with affective symptoms in major depressive disorder: a resting-state fMRI study. *Aust N Z J Psychiatry, 53*(6), 528-539. doi:10.1177/0004867419832106

Hu, L., Xiao, M., Ai, M., Wang, W., Chen, J., Tan, Z., . . . Kuang, L. (2019). Disruption of resting-state functional connectivity of right posterior insula in adolescents and young adults with major depressive disorder. *J Affect Disord, 257*, 23-30. doi:10.1016/j.jad.2019.06.057

Hu, Y., Zhao, C., Zhao, H., & Qiao, J. (2023). Abnormal functional connectivity of the nucleus accumbens subregions mediates the association between anhedonia and major depressive disorder. *BMC Psychiatry, 23*(1), 282. doi:10.1186/s12888-023-04693-0

Krug, S., Müller, T., Kayali, Ö., Leichter, E., Peschel, S., Jahn, N., . . . Sinke, C. (2022). Altered functional connectivity in common resting-state networks in patients with major depressive disorder: a resting-state functional connectivity study. *J Psychiatr Res, 155*, 33-41. doi:10.1016/j.jpsychires.2022.07.040

Lee, S., Lee, S. M., Kang, W. S., Jahng, G.-H., Ryu, C.-W., & Park, J. K. (2019). Altered resting-state functional connectivity in depressive disorder patients with suicidal attempts. *Neurosci Lett, 696*, 174-178. doi:10.1016/j.neulet.2018.12.037

Li, D., Zhang, H., Liu, Y., Liang, X., Chen, Y., Zheng, Y., . . . Cui, Y. (2021). Abnormal functional connectivity of posterior cingulate cortex correlates with phonemic verbal fluency deficits in major depressive disorder. *Front Neurol, 12*, 724874. doi:10.3389/fneur.2021.724874

Lin, Z., Xu, X., Wang, T., Huang, Z., & Wang, G. (2022). Abnormal regional homogeneity and functional connectivity in major depressive disorder patients with long-term remission: an exploratory study. *Psychiatry Res Neuroimaging, 327*, 111557. doi:10.1016/j.pscychresns.2022.111557

Liu, L., Zeng, L. L., Li, Y., Ma, Q., Li, B., Shen, H., & Hu, D. (2012). Altered cerebellar functional connectivity with intrinsic connectivity networks in adults with major depressive disorder. *PLoS One, 7*(6), e39516. doi:10.1371/journal.pone.0039516

Liu, Y., Chen, Y., Liang, X., Li, D., Zheng, Y., Zhang, H., . . . Qiu, S. (2020). Altered resting-state functional connectivity of multiple networks and disrupted correlation with executive function in major depressive disorder. *Front Neurol, 11*, 272. doi:10.3389/fneur.2020.00272

Long, J., Xu, J., Wang, X., Li, J., Rao, S., Wu, H., & Kuang, W. (2020). Altered local gyrification index and corresponding functional connectivity in medication free major depressive disorder. *J Frontiers in Psychiatry, 11*, 585401. doi:10.3389/fpsyt.2020.585401

Peng, D., Liddle, E. B., Iwabuchi, S. J., Zhang, C., Wu, Z., Liu, J., . . . Palaniyappan, L. (2015). Dissociated large-scale functional connectivity networks of the precuneus in medication-naive first-episode depression. *J Psychiatry Research: Neuroimaging, 232*(3), 250-256. doi:10.1016/j.pscychresns.2015.03.003

Penner, J., Osuch, E. A., Schaefer, B., Théberge, J., Neufeld, R. W. J., Menon, R. S., . . . Williamson, P. C. (2018). Temporoparietal Junction Functional Connectivity in Early Schizophrenia and Major Depressive Disorder. *Chronic Stress (Thousand Oaks), 2*, 2470547018815232. doi:10.1177/2470547018815232

Ramasubbu, R., Konduru, N., Cortese, F., Bray, S., Gaxiola-Valdez, I., & Goodyear, B. (2014). Reduced intrinsic connectivity of amygdala in adults with major depressive disorder. *Front Psychiatry, 5*, 17. doi:10.3389/fpsyt.2014.00017

Sawaya, H., Johnson, K., Schmidt, M., Arana, A., Chahine, G., Atoui, M., . . . Nahas, Z. (2015). Resting-state functional connectivity of antero-medial prefrontal cortex sub-regions in major depression and relationship to emotional intelligence. *Int J Neuropsychopharmacol, 18*(6). doi:10.1093/ijnp/pyu112

Tang, S., Li, H., Lu, L., Wang, Y., Zhang, L., Hu, X., . . . Huang, X. (2019). Anomalous functional connectivity of amygdala subregional networks in major depressive disorder. *Depress Anxiety, 36*(8), 712-722. doi:10.1002/da.22901

Tang, Y., Kong, L., Wu, F., Womer, F., Jiang, W., Cao, Y., . . . Wang, F. (2013). Decreased functional connectivity between the amygdala and the left ventral prefrontal cortex in treatment-naive patients with major depressive disorder: a resting-state functional magnetic resonance imaging study. *Psychol Med, 43*(9), 1921-1927. doi:10.1017/s0033291712002759

Todeva-Radneva, A., Kandilarova, S., Paunova, R., Stoyanov, D., Zdravkova, T., & Sladky, R. (2023). Functional Connectivity of the Anterior Cingulate Cortex and the Right Anterior Insula Differentiates between Major Depressive Disorder, Bipolar Disorder and Healthy Controls. *Biomedicines, 11*(6). doi:10.3390/biomedicines11061608

Wang, D., Xue, S. W., Tan, Z., Wang, Y., Lian, Z., & Sun, Y. (2019). Altered hypothalamic functional connectivity patterns in major depressive disorder. *Neuroreport, 30*(16), 1115-1120. doi:10.1097/wnr.0000000000001335

Wang, Y., Zhang, A., Yang, C., Li, G., Sun, N., Liu, P., . . . Zhang, K. (2020). Enhanced Functional Connectivity Within Executive Function Network in Remitted or Partially Remitted MDD Patients. *Front Psychiatry, 11*, 538333. doi:10.3389/fpsyt.2020.538333

Wang, Y. L., Yang, S. Z., Sun, W. L., Shi, Y. Z., & Duan, H. F. (2016). Altered functional interaction hub between affective network and cognitive control network in patients with major depressive disorder. *Behav Brain Res, 298*(Pt B), 301-309. doi:10.1016/j.bbr.2015.10.040

Wu, X., Lin, P., Yang, J., Song, H., Yang, R., & Yang, J. (2016). Dysfunction of the cingulo-opercular network in first-episode medication-naive patients with major depressive disorder. *J Affect Disord, 200*, 275-283. doi:10.1016/j.jad.2016.04.046

Wu, Z., Fang, X., Yu, L., Wang, D., Liu, R., Teng, X., . . . Zhang, C. (2022). Abnormal functional connectivity of the anterior cingulate cortex subregions mediates the association between anhedonia and sleep quality in major depressive disorder. *J Affect Disord, 296*, 400-407. doi:10.1016/j.jad.2021.09.104

Yang, X. H., Tian, K., Wang, D. F., Wang, Y., Cheung, E. F. C., Xie, G. R., & Chan, R. C. K. (2017). Anhedonia correlates with abnormal functional connectivity of the superior temporal gyrus and the caudate nucleus in patients with first-episode drug-naive major depressive disorder. *J Affect Disord, 218*, 284-290. doi:10.1016/j.jad.2017.04.053

Yang, Y., Zhong, N., Imamura, K., Lu, S., Li, M., Zhou, H., . . . Li, K. (2016). Task and Resting-State fMRI Reveal Altered Salience Responses to Positive Stimuli in Patients with Major Depressive Disorder. *PLoS One, 11*(5), e0155092. doi:10.1371/journal.pone.0155092

Ye, T., Peng, J., Nie, B., Gao, J., Liu, J., Li, Y., . . . Shan, B. (2012). Altered functional connectivity of the dorsolateral prefrontal cortex in first-episode patients with major depressive disorder. *Eur J Radiol, 81*(12), 4035-4040. doi:10.1016/j.ejrad.2011.04.058

Zhang, Q., Wu, J., Pei, C., Ma, M., Dong, Y., Gao, M., & Zhang, H. (2022). Altered functional connectivity in emotional subregions of the anterior cingulate cortex in young and middle-aged patients with major depressive disorder: A resting-state fMRI study. *Biol Psychol, 175*, 108426. doi:10.1016/j.biopsycho.2022.108426

Zhang, S., He, J. K., Zhong, G. L., Wang, Y., Zhao, Y. N., Wang, L., . . . Rong, P. J. (2022). Prolonged Longitudinal Transcutaneous Auricular Vagus Nerve Stimulation Effect on Striatal Functional Connectivity in Patients with Major Depressive Disorder. *Brain Sci, 12*(12). doi:10.3390/brainsci12121730

Zhang, X., Zhang, R., Lv, L., Qi, X., Shi, J., & Xie, S. (2022). Correlation between cognitive deficits and dorsolateral prefrontal cortex functional connectivity in first-episode depression. *J Affect Disord, 312*, 152-158. doi:10.1016/j.jad.2022.06.024

Zhou, B., Chen, Y., Zheng, R., Jiang, Y., Li, S., Wei, Y., . . . Cheng, J. (2022). Alterations of Static and Dynamic Functional Connectivity of the Nucleus Accumbens in Patients With Major Depressive Disorder. *Front Psychiatry, 13*, 877417. doi:10.3389/fpsyt.2022.877417

Zhou, Y., Yu, C., Zheng, H., Liu, Y., Song, M., Qin, W., . . . Jiang, T. (2010). Increased neural resources recruitment in the intrinsic organization in major depression. *J Affect Disord, 121*(3), 220-230. doi:10.1016/j.jad.2009.05.029

Zhu, J., Lin, X., Lin, C., Zhuo, C., & Yu, Y. (2018). Selective functional dysconnectivity of the dorsal-anterior subregion of the precuneus in drug-naive major depressive disorder. *J Affect Disord, 225*, 676-683. doi:10.1016/j.jad.2017.08.084

Zu, M., Wang, A., Bai, T., Xie, W., Guan, J., Tian, Y., & Wang, K. (2019). Resting-State Functional Connectivity Between Centromedial Amygdala and Insula as Related to Somatic Symptoms in Depressed Patients: A Preliminary Study. *Psychosom Med, 81*(5), 434-440. doi:10.1097/psy.0000000000000697

ICA studies (6 studies)

Dong, D., Ming, Q., Zhong, X., Pu, W., Zhang, X., Jiang, Y., . . . Yao, S. (2019). State-independent alterations of intrinsic brain network in current and remitted depression. *Prog Neuropsychopharmacol Biol Psychiatry, 89*, 475-480. doi:10.1016/j.pnpbp.2018.08.031

Lu, F., Cui, Q., Huang, X., Li, L., Duan, X., Chen, H., . . . Chen, H. (2020). Anomalous intrinsic connectivity within and between visual and auditory networks in major depressive disorder. *Prog Neuropsychopharmacol Biol Psychiatry, 100*, 109889. doi:10.1016/j.pnpbp.2020.109889

Luo, L., Wu, H., Xu, J., Chen, F., Wu, F., Wang, C., & Wang, J. (2021). Abnormal large-scale resting-state functional networks in drug-free major depressive disorder. *Brain Imaging Behav, 15*(1), 96-106. doi:10.1007/s11682-019-00236-y

Shi, H., Wang, X., Yi, J., Zhu, X., Zhang, X., Yang, J., & Yao, S. (2015). Default mode network alterations during implicit emotional faces processing in first-episode, treatment-naive major depression patients. *Front Psychol, 6*, 1198. doi:10.3389/fpsyg.2015.01198

Yu, R., Tan, H., Peng, G., Du, L., Wang, P., Zhang, Z., & Lyu, F. (2021). Anomalous functional connectivity within the default-mode network in treatment-naive patients possessing first-episode major depressive disorder. *Medicine (Baltimore), 100*(23), e26281. doi:10.1097/md.0000000000026281

Zhu, X., Wang, X., Xiao, J., Liao, J., Zhong, M., Wang, W., & Yao, S. (2012). Evidence of a dissociation pattern in resting-state default mode network connectivity in first-episode, treatment-naive major depression patients. *Biol Psychiatry, 71*(7), 611-617. doi:10.1016/j.biopsych.2011.10.035

Supplementary Tables

Table S1. The 10-point checklist of quality assessment for included studies.

| **Category 1: Participants** |
| --- |
| 1. Patients were evaluated according to specific diagnostic criteria, with demographic data and clinical characteristics reported. |
| 2. Comparison participants were evaluated, with psychiatric disorders and medical illnesses excluded. |
| 3. Important variables (e.g., age, sex, and education status) were controlled either by stratification or statistically. |
| 4. Sample size per group > 10. |
| **Category 2: Methods for image acquisition and analysis** |
| 5. Whole-brain seed-based functional connectivity or ICA analysis was performed. |
| 6. Coordinates were reported in a standard space (MNI or Talairach). |
| 7. A clear description of the MRI acquisition and preprocessing procedures was provided to facilitate replication. |
| 8. Measurements were clearly described to ensure reproducibility. |
| **Category 3: Results and conclusions** |
| 9. Statistical parameters for both significant and non-significant differences were provided. |
| 10. Conclusions were consistent with the results obtained, and the limitations were discussed. |

Note: The checklist was adapted from previous meta-analyses (Lin et al., 2023; Norman et al., 2016; Shepherd, Laurens, Matheson, Carr, & Green, 2012).

Abbreviations: ICA, independent component analysis; MNI, Montreal Neurological Institute.

Table S2. Quality assessment scores of included studies.

| Studies | 1 | 2 | 3 | 4 | 5 | 6 | 7 | 8 | 9 | 10 | Total |
| --- | --- | --- | --- | --- | --- | --- | --- | --- | --- | --- | --- |
| Beckmann et al. (2022) | 1 | 1 | 0.5 | 1 | 1 | 1 | 1 | 1 | 1 | 1 | 9.5 |
| Bessette et al. (2018) | 1 | 1 | 0.5 | 1 | 1 | 1 | 1 | 1 | 1 | 1 | 9.5 |
| Bluhm et al. (2009) | 1 | 1 | 0.5 | 1 | 1 | 1 | 1 | 1 | 1 | 1 | 9.5 |
| Cao et al. (2012) | 1 | 1 | 1 | 1 | 1 | 1 | 1 | 1 | 1 | 1 | 10 |
| Chen et al. (2021) | 1 | 1 | 1 | 1 | 1 | 1 | 1 | 1 | 1 | 1 | 10 |
| Chen et al. (2020) | 1 | 1 | 1 | 1 | 1 | 1 | 1 | 1 | 1 | 1 | 10 |
| Chen et al. (2015) | 0.5 | 1 | 0.5 | 1 | 1 | 1 | 1 | 1 | 1 | 1 | 9 |
| Crowther et al. (2015) | 1 | 1 | 0.5 | 1 | 1 | 1 | 1 | 1 | 1 | 1 | 9.5 |
| Cui et al. (2020) | 1 | 1 | 1 | 1 | 1 | 1 | 1 | 1 | 1 | 1 | 10 |
| Davey et al. (2012) | 1 | 1 | 0.5 | 1 | 1 | 1 | 1 | 1 | 1 | 1 | 9.5 |
| de Kwaasteniet et al. (2013) | 1 | 1 | 0.5 | 1 | 1 | 1 | 1 | 1 | 1 | 1 | 9.5 |
| de Kwaasteniet et al. (2015) | 1 | 1 | 0.5 | 1 | 1 | 1 | 1 | 1 | 1 | 1 | 9.5 |
| Dong et al. (2019) | 1 | 1 | 1 | 1 | 1 | 1 | 1 | 1 | 1 | 1 | 10 |
| Duan et al. (2020) | 0.5 | 1 | 0.5 | 1 | 1 | 1 | 1 | 1 | 1 | 1 | 9 |
| Fettes et al. (2018) | 0.5 | 1 | 0.5 | 1 | 1 | 1 | 1 | 1 | 1 | 1 | 9 |
| Frodl et al. (2010) | 1 | 1 | 0.5 | 1 | 1 | 1 | 1 | 1 | 1 | 1 | 9.5 |
| Furman et al. (2011) | 1 | 1 | 0.5 | 1 | 1 | 1 | 1 | 1 | 1 | 1 | 9.5 |
| Ge et al. (2019) | 1 | 1 | 0.5 | 1 | 1 | 1 | 1 | 1 | 1 | 1 | 9.5 |
| Guo et al. (2015a) | 1 | 1 | 1 | 1 | 1 | 1 | 1 | 1 | 1 | 1 | 10 |
| Guo et al. (2015b) | 1 | 1 | 1 | 1 | 1 | 1 | 1 | 1 | 1 | 1 | 10 |
| Hao et al. (2020) | 1 | 1 | 0.5 | 1 | 1 | 1 | 1 | 1 | 1 | 1 | 9.5 |
| He et al. (2019) | 1 | 1 | 0.5 | 1 | 1 | 1 | 1 | 1 | 1 | 1 | 9.5 |
| Hu et al. (2019) | 1 | 1 | 0.5 | 1 | 1 | 1 | 1 | 1 | 1 | 1 | 9.5 |
| Hu et al. (2023) | 1 | 1 | 0.5 | 1 | 1 | 1 | 1 | 1 | 1 | 1 | 9.5 |
| Krug et al. (2022) | 1 | 1 | 0.5 | 1 | 1 | 1 | 1 | 1 | 1 | 1 | 9.5 |
| Lee et al. (2019) | 0.5 | 1 | 0.5 | 1 | 1 | 1 | 1 | 1 | 1 | 1 | 9 |
| Li et al. (2021) | 1 | 1 | 0.5 | 1 | 1 | 1 | 1 | 1 | 1 | 1 | 9.5 |
| Lin et al. (2022) | 1 | 1 | 0.5 | 1 | 1 | 1 | 1 | 1 | 1 | 1 | 9.5 |
| Liu et al. (2012) | 1 | 1 | 0.5 | 1 | 1 | 1 | 1 | 1 | 1 | 1 | 9.5 |
| Liu et al. (2020) | 1 | 1 | 1 | 1 | 1 | 1 | 1 | 1 | 1 | 1 | 10 |
| Long et al. (2020) | 1 | 1 | 1 | 1 | 1 | 1 | 1 | 1 | 1 | 1 | 10 |
| Lu et al. (2020) | 1 | 1 | 1 | 1 | 1 | 1 | 1 | 1 | 1 | 1 | 10 |
| Luo et al. (2021) | 1 | 1 | 1 | 1 | 1 | 1 | 1 | 1 | 1 | 1 | 10 |
| Peng et al. (2015) | 1 | 1 | 1 | 1 | 1 | 1 | 1 | 1 | 1 | 1 | 10 |
| Penner et al. (2018) | 0.5 | 1 | 0.5 | 1 | 1 | 1 | 1 | 1 | 1 | 1 | 9 |
| Ramasubbu et al. (2014) | 1 | 1 | 0.5 | 1 | 1 | 1 | 1 | 1 | 1 | 1 | 9.5 |
| Sawaya et al. (2015) | 1 | 1 | 0.5 | 1 | 1 | 1 | 1 | 1 | 1 | 1 | 9.5 |
| Shi et al. (2015) | 0.5 |  | 0.5 | 1 | 1 | 1 | 1 | 1 | 1 | 1 | 9 |
| Tang et al. (2019) | 1 | 1 | 0.5 | 1 | 1 | 1 | 1 | 1 | 1 | 1 | 9.5 |
| Tang et al. (2013) | 1 | 1 | 1 | 1 | 1 | 1 | 1 | 1 | 1 | 1 | 10 |
| Todeva-Radneva et al. (2023) | 1 | 1 | 1 | 1 | 1 | 1 | 1 | 1 | 1 | 1 | 10 |
| Wang et al. (2019) | 1 | 1 | 0.5 | 1 | 1 | 1 | 1 | 1 | 1 | 1 | 9.5 |
| Wang et al. (2020) | 1 | 1 | 0.5 | 1 | 1 | 1 | 1 | 1 | 1 | 1 | 9.5 |
| Wang et al. (2016) | 1 | 1 | 1 | 1 | 1 | 1 | 1 | 1 | 1 | 1 | 10 |
| Wu et al. (2016) | 1 | 1 | 0.5 | 1 | 1 | 1 | 1 | 1 | 1 | 1 | 9.5 |
| Wu et al. (2022) | 1 | 1 | 1 | 1 | 1 | 1 | 1 | 1 | 1 | 1 | 10 |
| Yang et al. (2017) | 1 | 1 | 1 | 1 | 1 | 1 | 1 | 1 | 1 | 1 | 10 |
| Yang et al. (2016) | 1 | 1 | 0.5 | 1 | 1 | 1 | 1 | 1 | 1 | 1 | 9.5 |
| Ye et al. (2012) | 1 | 1 | 1 | 1 | 1 | 1 | 1 | 1 | 1 | 1 | 10 |
| Yu et al. (2021) | 1 | 1 | 0.5 | 1 | 1 | 1 | 1 | 1 | 1 | 1 | 9.5 |
| Zhang et al. (2022a) | 1 | 1 | 0.5 | 1 | 1 | 1 | 1 | 1 | 1 | 1 | 9.5 |
| Zhang et al. (2022b) | 1 | 1 | 0.5 | 1 | 1 | 1 | 1 | 1 | 1 | 1 | 9.5 |
| Zhang et al. (2022c) | 1 | 1 | 0.5 | 1 | 1 | 1 | 1 | 1 | 1 | 1 | 9.5 |
| Zhou et al. (2010) | 1 | 1 | 0.5 | 1 | 1 | 1 | 1 | 1 | 1 | 1 | 9.5 |
| Zhou et al. (2022) | 1 | 1 | 0.5 | 1 | 1 | 1 | 1 | 1 | 1 | 1 | 9.5 |
| Zhu et al. (2012) | 0.5 | 1 | 0.5 | 1 | 1 | 1 | 1 | 1 | 1 | 1 | 9 |
| Zhu et al. (2018) | 1 | 1 | 1 | 1 | 1 | 1 | 1 | 1 | 1 | 1 | 10 |
| Zu et al. (2019) | 1 | 1 | 0.5 | 1 | 1 | 1 | 1 | 1 | 1 | 1 | 9.5 |

Table S3. The demographic and clinical information of included studies.

| Studies | Sample size | | Age (y) | | Education (y) | | Gender (Female) | | Duration (y) | Diagnostic criteria | HAMD |
| --- | --- | --- | --- | --- | --- | --- | --- | --- | --- | --- | --- |
|  | Dep | HCs | Dep | HCs | Dep | HCs | Dep | HCs |  |  |  |
| Beckmann et al. (2022) | 27 | 36 | 31.00 | 31.80 | 15.50 | 15.50 | 0.63 | 0.64 | NA | DSM-IV | 21.70 |
| Bessette et al. (2018) | 47 | 35 | NA | NA | 14.70 | 14.50 | 0.72 | 0.60 | NA | DSM-IV-TR | 1.64 |
| Bluhm et al. (2009) | 14 | 15 | 21.90 | 23.50 | NA | NA | 0.64 | 0.73 | NA | DSM-IV | NA |
| Cao et al. (2012) | 42 | 32 | 29.20 | 26.10 | 12.70 | 13.50 | 0.57 | 0.47 | 13.11 | DSM-IV | 23.60 |
| Chen et al. (2021) | 62 | 61 | 27.40 | 24.70 | 13.90 | 13.70 | 0.63 | 0.53 | 1.89 | DSM-IV | 22.20 |
| Chen et al. (2020) | 40 | 20 | 46.50 | 46.00 | 10.40 | 11.10 | 0.48 | 0.60 | 2.06 | DSM-V | 26.23 |
| Chen et al. (2015) | 36 | 38 | 32.10 | 30.80 | 11.40 | 12.30 | 0.67 | 0.61 | NA | DSM-IV | 21.12 |
| Crowther et al. (2015) | 23 | 20 | 33.10 | 31.10 | NA | NA | 0.78 | 0.70 | 2.75 | DSM-IV | NA |
| Cui et al. (2020) | 23 | 21 | 44.90 | 38.70 | 11.60 | 12.80 | 0.70 | 0.71 | 5.62 | DSM-IV | 17.14 |
| Davey et al. (2012) | 18 | 20 | 18.90 | 19.90 | NA | NA | 0.67 | 0.60 | 0.88 | DSM-IV | NA |
| de Kwaasteniet et al. (2013) | 18 | 24 | 44.60 | 40.20 | NA | NA | 0.78 | 0.66 | NA | DSM-IV | 19.17 |
| de Kwaasteniet et al. (2015) | 17 | 18 | 52.50 | 51.50 | NA | NA | 0.53 | 0.56 | NA | DSM-IV | 21.80 |
| Dong et al. (2019) | 57 | 66 | 22.60 | 21.60 | 14.10 | 14.60 | 0.51 | 0.47 | 0.74 | DSM-IV-TR | 23.07 |
| Duan et al. (2020) | 29 | 29 | 28.70 | 26.80 | NA | NA | 0.69 | 0.48 | NA | DSM-V | 21.31 |
| Fettes et al. (2018) | 56 | 56 | 34.50 | 34.30 | NA | NA | 0.43 | 0.43 | NA | DSM-IV | 23.90 |
| Frodl et al. (2010) | 25 | 15 | 39.40 | 35.50 | NA | NA | 0.36 | 0.33 | 4.32 | DSM-IV | 20.06 |
| Furman et al. (2011) | 21 | 19 | 39.20 | 33.20 | NA | NA | 1.00 | 1.00 | NA | DSM-IV | NA |
| Ge et al. (2019) | 56 | 42 | 43.00 | 40.40 | 15.00 | 16.00 | 0.57 | 0.60 | NA | MINI | 22.09 |
| Guo et al. (2015a) | 44 | 44 | 27.50 | 29.40 | 12.50 | 12.10 | 0.50 | 0.55 | 1.63 | DSM-IV | 25.18 |
| Guo et al. (2015b) | 44 | 44 | 27.50 | 29.40 | 12.50 | 12.10 | 0.50 | 0.55 | 1.63 | DSM-IV | 25.18 |
| Hao et al. (2020) | 55 | 25 | 34.80 | 38.20 | 14.10 | 14.30 | 0.64 | 0.48 | NA | DSM-IV | 25.00 |
| He et al. (2019) | 34 | 34 | 36.20 | 34.80 | 12.60 | 12.80 | 0.59 | 0.59 | NA | DSM-IV | 28.88 |
| Hu et al. (2019) | 76 | 44 | 20.40 | 20.30 | 12.90 | 13.60 | 0.71 | 0.68 | NA | DSM-IV | 20.90 |
| Hu et al. (2023) | 55 | 30 | 35.20 | 36.80 | 11.50 | 11.90 | 0.67 | 0.70 | NA | DSM-V | NA |
| Krug et al. (2022) | 55 | 51 | 33.90 | 33.60 | NA | NA | 0.62 | 0.63 | NA | DSM-IV | NA |
| Lee et al. (2019) | 12 | 20 | 29.80 | 33.40 | 13.40 | 17.00 | 0.92 | 0.60 | NA | ICD-10 | NA |
| Li et al. (2021) | 34 | 34 | 29.40 | 30.10 | 13.00 | 13.70 | 0.74 | 0.71 | NA | DSM-V | 21.85 |
| Lin et al. (2022) | 34 | 30 | 37.70 | 35.80 | NA | NA | 0.59 | 0.70 | 5.44 | DSM-V | 7.15 |
| Liu et al. (2012) | 20 | 20 | 28.40 | 29.00 | 12.00 | 11.70 | 0.70 | 0.80 | 0.46 | DSM-IV | 26.10 |
| Liu et al. (2020) | 100 | 100 | 29.50 | 29.60 | 12.50 | 12.90 | 0.66 | 0.59 | 0.72 | DSM-V | 22.15 |
| Long et al. (2020) | 30 | 28 | 33.40 | 32.00 | 13.80 | 13.50 | 0.67 | 0.64 | 1.78 | DSM-IV | 31.20 |
| Lu et al. (2020) | 95 | 97 | 35.10 | 36.50 | 13.10 | 14.00 | 0.58 | 0.53 | 4.75 | DSM-IV-TR | 25.93 |
| Luo et al. (2021) | 27 | 54 | 29.70 | 29.40 | 13.80 | 13.80 | 0.63 | 0.63 | 3.24 | DSM-IV | 33.56 |
| Peng et al. (2015) | 16 | 16 | 34.40 | 33.80 | 15.60 | 15.80 | 0.56 | 0.56 | 0.15 | DSM-IV | 30.88 |
| Penner et al. (2018) | 24 | 24 | 21.20 | 23.80 | NA | NA | 0.67 | 0.50 | 1.13 | DSM-IV | NA |
| Ramasubbu et al. (2014) | 55 | 19 | 36.50 | 32.90 | 14.70 | 14.90 | 0.60 | 0.58 | NA | DSM-IV | 21..41 |
| Sawaya et al. (2015) | 21 | 21 | 37.30 | 38.30 | 3.57 | 6.62 | 0.81 | 0.81 | NA | DSM-IV | 23.29 |
| Shi et al. (2015) | 29 | 33 | 20.50 | 20.80 | 13.70 | 13.90 | 0.62 | 0.52 | NA | DSM-IV | NA |
| Tang et al. (2019) | 70 | 70 | 35.30 | 34.90 | NA | NA | 0.57 | 0.57 | 5.10 | DSM-IV | 23.24 |
| Tang et al. (2013) | 28 | 30 | 29.30 | 30.10 | 13.10 | 14.00 | 0.57 | 0.50 | 1.13 | DSM-IV | 29.00 |
| Todeva-Radneva et al. (2023) | 35 | 43 | 41.00 | 40.30 | 13.80 | 15.90 | 0.60 | 0.67 | 10.91 | DSM-IV-TR | NA |
| Wang et al. (2019) | 55 | 40 | 27.20 | 28.20 | NA | NA | 0.69 | 0.65 | NA | DSM-IV | 28.51 |
| Wang et al. (2020) | 19 | 17 | 32.00 | 30.50 | 12.00 | 11.80 | 0.58 | 0.47 | NA | DSM-IV | 21.37 |
| Wang et al. (2016) | 25 | 35 | 32.10 | 33.30 | 12.00 | 11.60 | 0.44 | 0.46 | 0.70 | DSM-IV | 29.32 |
| Wu et al. (2016) | 19 | 19 | 33.50 | 33.50 | 14.60 | 14.60 | 0.53 | 0.53 | NA | DSM-IV | 24.89 |
| Wu et al. (2022) | 41 | 63 | 25.60 | 27.20 | 14.00 | 14.20 | 0.68 | 0.60 | 0.55 | DSM-IV | 20.41 |
| Yang et al. (2017) | 40 | 36 | 28.80 | 29.10 | 13.90 | 13.50 | 0.47 | 0.53 | 0.51 | DSM-IV | 28.73 |
| Yang et al. (2016) | 19 | 19 | 33.80 | 33.30 | 14.10 | 13.90 | 0.58 | 0.58 | NA | DSM-IV | 15.80 |
| Ye et al. (2012) | 22 | 30 | 46.70 | 45.90 | 11.20 | 12.50 | 0.64 | 0.63 | 0.72 | DSM-IV | 18.50 |
| Yu et al. (2021) | 35 | 66 | 22.60 | 21.60 | 14.70 | 14.60 | 0.46 | 0.47 | 1.22 | DSM-IV | 25.08 |
| Zhang et al. (2022a) | 36 | 36 | 34.30 | 32.30 | 14.30 | 14.70 | 0.56 | 0.58 | NA | DSM-IV | 23.60 |
| Zhang et al. (2022b) | 15 | 16 | 40.90 | 35.50 | 14.30 | 14.70 | 0.80 | 0.56 | NA | DSM-V | 16.60 |
| Zhang et al. (2022c) | 37 | 53 | 26.00 | 24.50 | 14.40 | 14.70 | 0.57 | 0.49 | NA | DSM-IV | 24.24 |
| Zhou et al. (2010) | 18 | 20 | 38.90 | 40.60 | NA | NA | 0.78 | 0.70 | 0.58 | DSM-IV | 16.70 |
| Zhou et al. (2022) | 220 | 159 | 22.70 | 23.70 | 11.60 | 13.00 | 0.55 | 0.54 | NA | DSM-IV | 36.00 |
| Zhu et al. (2012) | 32 | 33 | 20.50 | 20.30 | NA | NA | 0.56 | 0.58 | 0.88 | DSM-IV | NA |
| Zhu et al. (2018) | 47 | 47 | 46.40 | 47.00 | 11.20 | 11.70 | 0.57 | 0.49 | 1.98 | DSM-IV | 30.30 |
| Zu et al. (2019) | 37 | 30 | 40.60 | 36.20 | NA | NA | 0.84 | 0.83 | 4.54 | DSM-IV | 26.35 |

Note: Age and education are presented as mean age (years) and mean education (years), respectively. Gender (female) represents the proportion of females in the total sample. Duration refers to the illness duration (years).

Abbreviations: Dep, depression; DSM-IV, Diagnostic and Statistical Manual of Mental Disorders, Fourth Edition; DSM-IV-TR, Diagnostic and Statistical Manual of Mental Disorders, Fourth Edition, Text Revision; DSM-V, Diagnostic and Statistical Manual of Mental Disorders, Fifth Edition; HAMD, Hamilton Depression Rating Scale; HCs, healthy controls; ICD-10, International Classification of Diseases, Tenth Revision; MINI, Mini-International Neuropsychiatric Interview; NA, not available; y, year.

Table S4. The MRI acquisition and preprocessing details of included studies.

| Studies | Scanner (FS, T) | TE (ms) | TR (ms) | Check whole brain coverage | Methodology | Head motion | | GSR | Voxel size (mm^3^) | |
| --- | --- | --- | --- | --- | --- | --- | --- | --- | --- | --- |
|  |  |  |  |  |  | Realign | Regression |  | Scan | Normalization |
| Beckmann et al. (2022) | Philips (3T) | 28 | 2000 | Y | Seed-based | Y | Y | Y | 3×3×3 | 3×3×3 |
| Bessette et al. (2018) | GE (3T) | 30 | 2000 | N | Seed-based | Y | Y | N | NA | 3×3×3 |
| Bluhm et al. (2009) | Siemens (4T) | 15 | 4000 | N | Seed-based | Y | N | N | 4×4×4 | NA |
| Cao et al. (2012) | Siemens (3T) | 30 | 2000 | N | Seed-based | Y | Y | Y | NA | 3×3×3 |
| Chen et al. (2021) | Siemens (3T) | 30 | 2000 | Y | Seed-based | Y | Y | N | 4×4×5 | 3×3×3 |
| Chen et al. (2020) | Siemens (1.5T) | 25 | 2000 | Y | Seed-based | Y | Y | Y | 3.75×3.75×4 | 2×2×2 |
| Chen et al. (2015) | Siemens (3T) | 40 | 3000 | N | Seed-based | Y | Y | Y | NA | 3×3×3 |
| Crowther et al. (2015) | GE (3T) | 30 | 1500 | Y | Seed-based | Y | Y | N | 3.75×3.75×4 | NA |
| Cui et al. (2020) | GE (3T) | 30 | 2000 | N | Seed-based | Y | Y | N | NA | 3×3×3 |
| Davey et al. (2012) | Siemens (3T) | 40 | 2400 | N | Seed-based | Y | N | Y | NA | 2×2×2 |
| de Kwaasteniet et al. (2013) | Philips (3T) | 25 | 2300 | N | Seed-based | Y | Y | N | 3×2.29×2.29 | NA |
| de Kwaasteniet et al. (2015) | Philips (3T) | 30 | 2300 | N | Seed-based | Y | Y | N | 3×2.29×2.29 | 4×4×4 |
| Dong et al. (2019) | Siemens (3T) | 30 | 2000 | N | ICA | Y | N | N | NA | 3×3×3 |
| Duan et al. (2020) | Siemens (3T) | 30 | 2000 | N | Seed-based | Y | Y | N | NA | 3×3×3 |
| Fettes et al. (2018) | GE (3T) | 30 | 2000 | N | Seed-based | Y | Y | N | NA | 2×2×2 |
| Frodl et al. (2010) | GE (3T) | 35 | 2100 | Y | Seed-based | Y | N | N | NA | NA |
| Furman et al. (2011) | GE (3T) | 30 | 1200 | N | Seed-based | Y | Y | Y | 3.44×3.44×5 | 3×3×3 |
| Ge et al. (2019) | Philips (3T) | 30 | 2000 | N | Seed-based | Y | Y | N | 2.75×2.75×5 | 3×3×3 |
| Guo et al. (2015a) | Siemens (3T) | 30 | 2000 | N | Seed-based | Y | Y | N | NA | 3×3×3 |
| Guo et al. (2015b) | Siemens (3T) | 30 | 2000 | N | Seed-based | Y | Y | N | NA | 3×3×3 |
| Hao et al. (2020) | Siemens (3T) | 40 | 3000 | N | Seed-based | Y | Y | Y | NA | 3×3×3 |
| He et al. (2019) | GE (3T) | 30 | 2000 | N | Seed-based | Y | Y | N | 3.75×3.75×3.2 | 3×3×3 |
| Hu et al. (2019) | GE (3T) | 40 | 2000 | N | Seed-based | Y | Y | N | NA | NA |
| Hu et al. (2023) | Siemens (3T) | 2.58 | 1900 | N | Seed-based | Y | Y | N | 3×3×3 | 4×4×4 |
| Krug et al. (2022) | Siemens (3T) | 36 | 1310 | N | Seed-based | Y | Y | Y | 2×2×2 | 2×2×2 |
| Lee et al. (2019) | Philips (3T) | 35 | 3000 | N | Seed-based | Y | Y | N | 2.88×2.91×4.00 | NA |
| Li et al. (2021) | GE (3T) | 30 | 2000 | N | Seed-based | Y | Y | N | NA | NA |
| Lin et al. (2022) | Siemens (3T) | 4.1 | 2000 | N | Seed-based | Y | Y | N | 0.9×1.2×1.2 | 3×3×3 |
| Liu et al. (2012) | GE (1.5T) | 50 | 2000 | N | Seed-based | Y | Y | Y | NA | NA |
| Liu et al. (2020) | GE (3T) | 30 | 2000 | N | Seed-based | Y | Y | N | NA | NA |
| Long et al. (2020) | Philips (3T) | 30 | 2000 | N | Seed-based | Y | Y | Y | 3.44×3.44×4.6 | 3×3×3 |
| Lu et al. (2020) | GE (3T) | 30 | 2000 | N | ICA | Y | N | N | 3.7×3.75×3.2 | 3×3×3 |
| Luo et al. (2021) | Philips (3T) | 30 | 2000 | N | ICA | Y | N | N | NA | 3×3×3 |
| Peng et al. (2015) | GE (3T) | 30 | 3000 | N | Seed-based | Y | Y | Y | 3.75×3.75×5.0 | 3×3×3 |
| Penner et al. (2018) | Siemens (3T) | 20 | 3000 | N | Seed-based | Y | Y | N | 2×2×2 | NA |
| Ramasubbu et al. (2014) | GE (3T) | 30 | 2000 | N | Seed-based | Y | Y | N | NA | NA |
| Sawaya et al. (2015) | Siemens (3T) | 35 | 2410 | N | Seed-based | Y | Y | Y | 3×3×3 | NA |
| Shi et al. (2015) | Siemens (1.5T) | 40 | 3000 | N | ICA | Y | N | N | 3.8×3.8×4 | 2×2×2 |
| Tang et al. (2019) | GE (3T) | 30 | 2000 | N | Seed-based | Y | Y | N | 3.75×3.75×5 | 3×3×3 |
| Tang et al. (2013) | GE (3T) | 40 | 2000 | N | Seed-based | Y | Y | Y | NA | 3×3×3 |
| Todeva-Radneva et al. (2023) | GE (3T) | 30 | 2000 | N | Seed-based | Y | Y | N | NA | NA |
| Wang et al. (2019) | GE (3T) | 22 | 2000 | N | Seed-based | Y | Y | N | 2.5×2.5×2.5 | 3×3×3 |
| Wang et al. (2020) | Siemens (3T) | 30 | 2000 | N | Seed-based | Y | Y | Y | NA | 3×3×3 |
| Wang et al. (2016) | Siemens (3T) | 25 | 2000 | N | Seed-based | Y | Y | Y | 3.75×3.75×4 | 2×2×2 |
| Wu et al. (2016) | GE (1.5T) | 35 | 2500 | N | Seed-based | Y | Y | Y | 4×4×4 | NA |
| Wu et al. (2022) | Siemens (3T) | 30 | 800 | N | Seed-based | Y | Y | N | NA | 2×2×2 |
| Yang et al. (2017) | Siemens (3T) | 25 | 2000 | N | Seed-based | Y | Y | Y | NA | 3×3×3 |
| Yang et al. (2016) | Siemens (3T) | 31 | 2000 | N | Seed-based | Y | N | Y | NA | 3×3×3 |
| Ye et al. (2012) | Siemens (3T) | 40 | 2000 | N | Seed-based | Y | N | N | NA | 3×3×3 |
| Yu et al. (2021) | Siemens (3T) | 30 | 2000 | N | ICA | Y | N | Y | NA | 3×3×3 |
| Zhang et al. (2022a) | GE (1.5T) | 35 | 2500 | N | Seed-based | Y | Y | Y | NA | NA |
| Zhang et al. (2022b) | Siemens (3T) | 30 | 2000 | N | Seed-based | Y | Y | N | NA | 3×3×3 |
| Zhang et al. (2022c) | Siemens (3T) | 30 | 2500 | N | Seed-based | Y | Y | Y | NA | 3×3×3 |
| Zhou et al. (2010) | Siemens (3T) | 30 | 2000 | N | Seed-based | Y | Y | N | NA | 3×3×3 |
| Zhou et al. (2022) | GE (3T) | 40 | 2000 | N | Seed-based | Y | Y | N | NA | 3×3×3 |
| Zhu et al. (2012) | Siemens (1.5T) | 40 | 2000 | N | Seed-based | Y | N | N | 5×3.85×3.85 | 3×3×3 |
| Zhu et al. (2018) | Siemens (3T) | 25 | 2000 | N | ICA | Y | Y | Y | NA | 3×3×3 |
| Zu et al. (2019) | GE (3T) | 30 | 2400 | N | Seed-based | Y | Y | N | 3×3×3 | 3×3×3 |

Note: Check whole brain coverage indicates whether whole-brain coverage was verified during image acquisition. Head motion refers to the control and management of head movement during realignment or regression in preprocessing. “Y” indicates that the step was performed, while “N” indicates it was not performed or the information is unavailable. “Scan” refers to the voxel size during the initial MRI scan acquisition, while “Normalization” refers to the voxel size after spatial normalization to a standard stereotaxic space (e.g., MNI or Talairach).

Abbreviations: FS, field strength; GSR, global signal regression; ICA, independent component analysis; MNI, Montreal Neurological Institute; NA, not available; T, Tesla; TE, echo time; TR, repetition time.

Table S5. The statistical overview of included studies.

| Studies | Software | Statistical methods | Thresholding strategy | Study-specific details |
| --- | --- | --- | --- | --- |
| Beckmann et al. (2022) | SPM | ANOVA + Post-hoc *t*-tests | Cluster-level correction (FWE)  Uncorrected voxel *p* < 0.001, corrected cluster *p* < 0.05 | All clusters were formed using a cluster-forming-threshold of *p* < .001 and controlled for Family-Wise-Error (*p*FWE-corr < 0.05). |
| Bessette et al. (2018) | SPM | ANCOVA+ Post-hoc *F*-tests | Cluster-level correction (FWE)  Uncorrected voxel *p* < 0.005, corrected cluster *p* < 0.04 | Bayesian whole-brain correction of *p* = 0.01 is achieved with a joint threshold of height and extent (*k* > 57, *p* < 0.005, 440 mm^3^) for each seed-based *F*-test analysis for a family-wise error rate for 4 analyses at *p* < 0.04. |
| Bluhm et al. (2009) | SPM | Mixed-effects model | Voxel-level correction (FDR)  FDR *p* < 0.001 | Reported within-group findings reflect a type 1 error threshold of *P* < 0.001 using false discovery rate correction for multiple comparisons. |
| Cao et al. (2012) | FSL | Two-sample *t*-tests | Cluster-level correction (GRF)  Uncorrected voxel *p* < 0.01, corrected cluster *p* < 0.025 | Given that our analyses were done for left and right hippocampal-ROIs respectively, we set a cluster level threshold of *p* < 0.025, corrected (0.05/2), with a minimum *Z* > 2.3 and a voxel level threshold of *p* < 0.01, uncorrected. |
| Chen et al. (2021) | SPM | Two-sample *t*-tests | Voxel-level correction (FDR)  FDR *p* < 0.05 | Statistical parametric maps were generated after multiple comparison analyses with a false discovery rate *p* < 0.05 and cluster size > 20. |
| Chen et al. (2020) | DPABI | Two-sample *t*-tests | Voxel-level correction (FDR)  FDR *p* < 0.01 | The statistical maps were managed by False Discovery Rate (FDR) for multiple comparisons to a significant level of *p* < 0.01, with cluster size over 160 mm^3^. |
| Chen et al. (2015) | SPM | Two-sample *t-*tests | Voxel-level correction (FDR)  FDR *p* < 0.05 | The significance level was set at *P* < 0.05, and was corrected for multiple comparisons using FDR-criterion with cluster size > 540 mm^3^. |
| Crowther et al. (2015) | FSL | Two-sample *t*-tests | Cluster-level correction (GRF)  Uncorrected voxel *p* < 0.01, corrected cluster *p* < 0.05 | Using *Z*-statistic images cluster thresholded at *Z* > 2.3 with a corrected cluster significance threshold of *p* < 0.05. |
| Cui et al. (2020) | DPABI | Two-sample *t*-tests | Cluster-level correction (FWE)  Uncorrected voxel *p* < 0.001, corrected cluster *p* < 0.01 | Seed-to-voxel results were reported when significant at a cluster-level threshold of *p* < 0.01 false discovery rate (FDR) corrected and a voxel-level threshold of *p* < 0.001 uncorrected. |
| Davey et al. (2012) | SPM | Two-sample *t*-tests | Cluster-level correction (FWE)  Uncorrected voxel *p* < 0.001, corrected cluster *p* < 0.05 | resulting 2-transformed statistical maps were thresholded at *p* < 0.001, with cluster-wise family wise error (FWE) correction of *p*FWE < 0.05 for the whole brain volume. |
| de Kwaasteniet et al. (2013) | SPM | Two-sample *t*-tests | Voxel-level correction (FWE)  Corrected cluster *p* < 0.05 | We used voxel-wise statistical tests with family-wise error (FWE) correction (*p* < 0.05) for multiple comparisons across the entire brain or with ROIs with small volume corrections. |
| de Kwaasteniet et al. (2015) | SPM | Two-sample *t*-tests | Cluster-level correction (FWE)  Uncorrected voxel *p* < 0.005, corrected cluster *p* < 0.05 | All statistical tests were family wise error (FWE) rate corrected (*p* < 0.05) for multiple comparisons at the cluster level (using a cluster-forming threshold of *p* < 0.005 uncorrected) across the entire brain. |
| Dong et al. (2019) | GIFT | ANOVA + Post-hoc *t*-tests | Voxel-level correction (FDR)  Uncorrected voxel *p* < 0.005,  FDR *p* < 0.05 | All imaging results were corrected via false discovery rate (FDR) correction surpassing a *p* < .005 initial voxel threshold (significance at *p* < 0.05). |
| Duan et al. (2020) | SPM | Two-sample *t*-tests | Cluster-level correction (FDR)  Uncorrected voxel *p* < 0.005, corrected cluster *p* < 0.05 | The contrast map was thresholded at a voxel-level threshold of 0.005 with a cluster-level threshold of 0.05 (false discovery rate (FDR) corrected). |
| Fettes et al. (2018) | CONN | Mixed-effect general linear models | Cluster-level correction (FDR)  Uncorrected voxel *p* < 0.05, corrected cluster *p* < 0.01 | Resulting group-level analyses were thresholded at a *p* < .05 (cluster corrected using the false discovery rate with a height threshold of *p* < .01). |
| Frodl et al. (2010) | SPM | ANCOVA + Post-hoc *t*-tests | Voxel-level correction (FWE)  Corrected cluster p < 0.05  Or  Cluster-level correction (FWE)  Uncorrected voxel *p* < 0.001, corrected cluster *p* < 0.05 | Statistical significance was based on a threshold of *p* < .05 (family-wise error [FWE], voxel-level corrected). Moreover, cluster-level statistical analyses were performed and are also reported in the tables (cluster level, *p* < .05, FWE corrected with a primary threshold of *p* < .001). |
| Furman et al. (2011) | AFNI | Two-sample *t*-tests | Cluster-level correction (AlphaSim)  Uncorrected voxel *p* < 0.001, corrected cluster *p* < 0.05 | AlphaSim and determined that an uncorrected single voxel significant threshold of *p* < 0.005 and cluster threshold of *k* = 26 were necessary to hold family-wise type I error at *P* < 0.05. |
| Ge et al. (2019) | SPM | Two-sample *t*-tests | Cluster-level correction (FWE)  Uncorrected voxel *p* < 0.005, corrected cluster *p* < 0.008 | The significance level for each comparison was set to α< 0.05/6 = 0.008 (Bonferroni correction was applied here to correct for multiple comparisons of the 6 subregions), with a cluster level family-wise error (FWE) correction and height threshold of *p* < 0.005. |
| Guo et al. (2015a) | REST | Two-sample *t*-tests | Cluster-level correction (GRF)  Uncorrected voxel *p* < 0.005, corrected cluster *p* < 0.005 | The significance level for each group was set at *P* < 0.005 (GRF corrected, min *z* > 2.807, cluster significance: *P* < 0.005). |
| Guo et al. (2015b) | REST | Two-sample *t*-tests | Cluster-level correction (GRF)  Uncorrected voxel *p* < 0.001, corrected cluster *p* < 0.001 | The significance level for each group was set at *p* < 0.001 (GRF corrected, min *z* > 3.2905, cluster significance: *p* < 0.001). |
| Hao et al. (2020) | SPM | Two-sample *t*-tests | Cluster-level correction (AlphaSim)  Uncorrected voxel *p* < 0.001, corrected cluster *p* < 0.05 | Results were corrected using the AlphaSim method (threshold of *P* < 0.001, cluster: *p* < 0.05). |
| He et al. (2019) | REST | Two-sample *t*-tests | Cluster-level correction (AlphaSim)  Uncorrected voxel *p* < 0.001, corrected cluster *p* < 0.05 | The multiple comparison correction was conducted at a cluster level of *p* < 0.05 by employing AlphaSim program in the REST toolbox with a height threshold of *p* < 0.001. |
| Hu et al. (2019) | DPABI | Permutation tests | TFCE-level correction (FWE)  Corrected *p* < 0.001 | The significance level was set at *p* < 0.05 with 1000 permutations corrected with TFCE correction. |
| Hu et al. (2023) | CONN | Two-sample *t*-tests | Cluster-level correction (FWE)  Uncorrected voxel *p* < 0.001, corrected cluster *p* < 0.05 | Thresholded at a whole brain *P* < 0.001 uncorrected voxel threshold and cluster level FWE *p* < 0.05. |
| Krug et al. (2022) | SPM | Two-sample *t-*tests | Cluster-level correction (FWE)  Unknown voxel *p* threshold,  corrected cluster *p* < 0.05 | Results were considered significant when *p* < 0.05 using family wise error (FWE) correction for multiple comparisons on a cluster level. All *p* values remain significant after applying Bonferroni correction for 8 tests (tests (n) = 8, desired α = 0.05, α/n = 0.05/8 = 0.00625). |
| Lee et al. (2019) | NA | ANCOVA + Post-hoc *t*-tests | Cluster-level correction (FDR)  Uncorrected voxel *p* < 0.001, corrected cluster *p* < 0.05 | The threshold at the voxel level was *p* < 0.001, which was then corrected for whole brain comparisons by FDR to *p* < 0.05. |
| Li et al. (2021) | SPM | Two-sample *t*-tests | Cluster-level correction (FWE)  Uncorrected voxel *p* < 0.001, corrected cluster *p* < 0.05 | We used *P* < 0.001 for the cluster-forming threshold and implemented a family-wise error (FWE) correction approach at the cluster level. All results survived whole-brain cluster correction (*P*_FWE_ < 0.05). |
| Lin et al. (2022) | SPM | Two-sample *t*-tests | Cluster-level correction (AlphaSim)  Corrected cluster *p* < 0.05 | AlphaSim correction was applied for each test to adjust multiple tests (*P* < 0.01, AlphaSim correction, cluster size >50 voxels). |
| Liu et al. (2012) | SPM | Two-sample *t*-tests | Voxel-level correction (FDR)  FDR *p* < 0.05  Or  Uncorrected voxel *p* < 0.001 | The resulting statistical maps were set at a combined threshold of *p* < 0.05 (False Discovery Rate corrected) with cluster size > 10 voxels. For the maps which did not meet this criterion, a relaxed threshold of *p* < 0.001 (uncorrected) with cluster size > 50 voxels was also used in this study. |
| Liu et al. (2020) | SPM | Two-sample *t*-tests | Cluster-level correction (FWE)  Uncorrected voxel *p* < 0.001, corrected cluster *p* < 0.05 | We used *P* < 0.001 for the cluster-forming threshold and implemented a familywise error (FWE) correction approach at the cluster level. All results survived whole-brain cluster correction (*P*_FWE_ < 0.05). |
| Long et al. (2020) | NA | Two-sample *t*-tests | Cluster-level correction (AlphaSim)  Uncorrected voxel *p* < 0.001, corrected cluster *p* < 0.05 | AlphaSim corrected threshold of *p* < 0.05 (cluster-forming threshold at voxel-level *p* < 0.001, and minimum cluster size = 56). |
| Lu et al. (2020) | GIFT | Two-sample *t-*tests | Cluster-level correction (GRF)  Uncorrected voxel *p* < 0.05, corrected cluster *p* < 0.05 | The multiple comparisons were corrected by using the Gaussian random field (GRF) theory with a cluster-defining threshold (CDT) of *P* = .05, voxel *Z* > 1.96, cluster level *p* < .05. |
| Luo et al. (2021) | GIFT | Two-sample *t*-tests | Cluster-level correction (AlphaSim)  Uncorrected voxel *p* < 0.001, corrected cluster *p* < 0.05 | A cluster-level Monte Carlo simulation (5000 times) corrected threshold of *p* < 0.05 was used to identify the significant changes in functional connectivities (cluster forming threshold at voxel-level *p* < 0.001). |
| Peng et al. (2015) | SPM | Two-sample *t*-tests | Cluster-level correction (FWE)  Uncorrected voxel *p* < 0.05, corrected cluster *p* < 0.05 | Based on a voxel inclusion threshold of *p* < 0.05, uncorrected, we report only clusters of significant voxels large enough to survive FWE correction for multiple testing at *p* < 0.05. |
| Penner et al. (2018) | NA | NA | Cluster-level correction (FWE)  Uncorrected voxel *p* < 0.001, corrected cluster *p* < 0.05 | Statistical threshold set at cluster size *k* > 100 and *p* < 0.001 (uncorrected), cluster *p* value (*p* < 0.05, FWE-corrected). |
| Ramasubbu et al. (2014) | FSL | Mixed-effects model | Cluster-level correction (AlphaSim)  Uncorrected voxel *p* < 0.01, corrected cluster *p* < 0.05 | From these analyses, *Z*-statistic images were generated with a statistical threshold of *Z* < 2.3 (*p* < 0.01), and a cluster size threshold of at least 77 contiguous voxels corresponding to a corrected *p*-value of 0.05 as determined by Monte Carlo simulations using AlphaSim. |
| Sawaya et al. (2015) | FSL | Mixed-effects model | Cluster-level correction (GRF)  Uncorrected voxel *p* < 0.01, corrected cluster *p* < 0.05 | All statistical maps were thresholded at *Z* > 2.3 with a cluster-corrected significance threshold of *p* < 0.05, using random field analysis. |
| Shi et al. (2015) | GIFT | Two-sample *t*-tests | Cluster-level correction (FWE)  Uncorrected voxel *p* < 0.001, corrected cluster *p* < 0.05 | For the second-level analysis, thresholds were set at voxel-level uncorrected *p* < 0.001 and cluster-level family-wise error (FWE)-corrected *p* < 0.05. |
| Tang et al. (2019) | SPM | Two-sample *t*-tests | Cluster-level correction (AlphaSim)  Uncorrected voxel *p* < 0.001, corrected cluster *p* < 0.05 | Therefore, the combination criteria were determined by the AlphaSim program at a single voxel threshold of *p* < 0.001 and cluster size >18 voxels (486 mm^3^), corresponding to a corrected *p* < 0.05. |
| Tang et al. (2013) | SPM | Two-sample *t*-tests | Cluster-level correction (FWE)  Uncorrected voxel *p* < 0.005, corrected cluster *p* < 0.05 | To control the false positive rates, we set the significance threshold to *p* < 0.005 at the voxel level and corrected it for multiple comparisons using cluster‐level family‐wise error (FWE) thresholding (*p* < 0.05). |
| Todeva-Radneva et al. (2023) | CONN | Two-sample *t*-tests | Cluster-level correction (FWE)  Uncorrected voxel *p* < 0.001, corrected cluster *p* < 0.05 | Cluster-level inferences on the between-group-level parametric statistics were based on random field theory with a voxel threshold *p* < 0.001 (uncorrected) and a cluster threshold *p* < 0.05 (cluster-size family-wise error (FWE) corrected). |
| Wang et al. (2019) | NA | Two-sample *t-*tests | Cluster-level correction (GRF)  Uncorrected voxel *p* < 0.001, corrected cluster *p* < 0.05 | A multiple comparison correction was performed using Gaussian random field theory (GRF) with voxel *P* < 0.001 and a GRF cluster-corrected threshold of *P* < 0.05. |
| Wang et al. (2020) | DPABI | NA | Cluster-level correction (GRF)  Uncorrected voxel *p* < 0.001,  Unknown cluster *p* threshold | Gaussian Random Field (GRF) Theory correction was used for multiple comparison correction with voxel *p* < 0.001. |
| Wang et al. (2016) | SPM | GLM | Voxel-level correction (FDR)  FDR *p* < 0.05 | Multiple comparison correction was performed using FDR-corrected *p* < 0.05 and cluster extent *k* > 100 voxels (800 mm^3^). |
| Wu et al. (2016) | NA | Two-sample *t*-tests | Uncorrected *p* < 0.001 | Two-sample *t*-test (*p* < 0.001, uncorrected). |
| Wu et al. (2022) | DPABI | Two-sample *t*-tests | Cluster-level correction (GRF)  Uncorrected voxel *p* < 0.001, corrected cluster *p* < 0.05 | Gaussian Random Field (GRF) correction was used to control for multiple comparisons, with a voxel level of *p* < 0.001 and cluster level of *p* < 0.05 (two-tailed). |
| Yang et al. (2017) | DPABI | Two-sample *t*-tests | Cluster-level correction (AlphaSim)  Uncorrected voxel *p* < 0.001, corrected cluster *p* < 0.05 | Statistical maps were set at a combined threshold of *p* < 0.001 and a minimum cluster size of 33 voxels, yielding a threshold of *p* < 0.05 AlphaSim corrected. |
| Yang et al. (2016) | REST | Two-sample *t*-tests | Cluster-level correction (AlphaSim)  Uncorrected voxel *p* < 0.005, corrected cluster *p* < 0.05 | Thresholds were set at a voxel-level *p* < 0.005, cluster size > 1242 mm^3^, corresponding to a corrected *p* < 0.05 as determined by AlphaSim correction. |
| Ye et al. (2012) | SPM | Two-sample *t*-tests | Uncorrected *p* < 0.005 | Brain regions with significant changes of correlation coefficients were yielded at the voxel-level with a height threshold of *p* < 0.005 (uncorrected) and a cluster-extent threshold of 20 voxels. |
| Yu et al. (2021) | REST | Two-sample *t*-tests | Cluster-level correction (GRF)  Uncorrected voxel *p* < 0.01, corrected cluster *p* < 0.01 | According to the theory of Gaussian random field, a *P* value of less than .01 shows a considerable statistical difference between the multiple comparisons. Through voxel significance, the needs are met at *P* < .01, and cluster implication for *P* < .01. |
| Zhang et al. (2022a) | NA | Two-sample *t*-tests | Voxel-level correction (FDR)  FDR *p* < 0.05  Or  Cluster-level correction (AlphaSim)  Uncorrected voxel *p* < 0.001, corrected cluster *p* < 0.01 | Two sample *t*-test was applied to examine the group differences of ACC-related FC (*p* < 0.05, false discovery rate (FDR) corrected). The cluster threshold was 0.001, and 10000 Monte Carlo simulations were applied to correct for multiple comparisons across surfaces using FreeSurfer. The Monte Carlo analysis determined the likelihood that the resulting clusters were fully corrected for multiple comparisons across surfaces. The corrected *p* value for clusters was set to *p* < 0.01, and the cluster size was set to 200 mm^2^. |
| Zhang et al. (2022b) | NA | Two-sample *t*-tests | Cluster-level correction (GRF)  Uncorrected voxel *p* < 0.001, corrected cluster *p* < 0.05 | (*p* < 0.001 at the voxel level and *p* < 0.05 at the cluster level, two-sided test) |
| Zhang et al. (2022c) | DPABI | Two-sample *t*-tests | Cluster-level correction (AlphaSim)  Uncorrected voxel *p* < 0.05, corrected cluster *p* < 0.01 | The results were corrected for multiple comparisons using the AlphaSim program. A cluster-corrected *p* < 0.01 (voxel level *p* < 0.05, cluster size > 50 voxels). |
| Zhou et al. (2010) | AFNI | Two-sample *t*-tests | Cluster-level correction (AlphaSim)  Uncorrected voxel *p* < 0.01, corrected cluster *p* < 0.001 | A combined threshold of contrast maps was set at *p* < 0.01 for each voxel and a cluster size of at least 675 mm^3^, which was equal to the corrected threshold of *p* < 0.001, determined by a Monte Carlo simulation. |
| Zhou et al. (2022) | REST | Two-sample *t*-tests | Cluster-level correction (GRF)  Uncorrected voxel *p* < 0.001, corrected cluster *p* < 0.05 | All results reported were thresholded using the Gaussian random field (GRF) correction with a voxel level threshold of *p* < 0.001 and a cluster-level threshold of *p* < 0.05. |
| Zhu et al. (2012) | REST | Two-sample *t*-tests | Cluster-level correction (AlphaSim)  Uncorrected voxel *p* < 0.01, corrected cluster *p* < 0.05 | The statistical results were corrected using AlphaSim program in the Resting-State fMRI Data Analysis Toolkit (REST) software (Forum of resting-state fMRI, http://restfmri.net/forum/index.php) at .05 significance level (combined height threshold *p* < .01 and a minimum cluster size > 33. |
| Zhu et al. (2018) | NA | Two-sample *t*-tests | Voxel-level correction (FDR)  FDR *p* < 0.05 | Multiple comparisons for these analyses were corrected using a voxel-level false discovery rate (FDR) method with a corrected threshold of *P* < 0.05. |
| Zu et al. (2019) | NA | Two-sample *t*-tests | Cluster-level correction (GRF)  Uncorrected voxel *p* < 0.0005, corrected cluster *p* < 0.05 | All statistical maps were corrected with a Gaussian Random Field method with the significance of voxel level set at *p* < .0005, and that of cluster level set at *p* < .05, two-tailed. |

Abbreviations: ANCOVA, Analysis of Covariance; ANOVA, Analysis of Variance; GLM, General Linear Model; NA, not available.

Table S6. Summary of seed-networks and anatomical regions of included studies in this meta-analysis.

| Studies | Seed-networks | | | | | | | | | | | | | |
| --- | --- | --- | --- | --- | --- | --- | --- | --- | --- | --- | --- | --- | --- | --- |
|  | LN | | DMN | | VN | | VAN | | DAN | | FPN | | SMN | |
|  | W | B | W | B | W | B | W | B | W | B | W | B | W | B |
| Beckmann et al. (2022) |  |  |  |  |  |  |  | ACC |  | FFG |  | APFC DMPFC |  |  |
| Bessette et al. (2018) |  |  | PCC Hippo | PCC Hippo |  |  |  |  |  |  |  |  |  |  |
| Bluhm et al. (2009) |  |  | PCC | PCC |  |  |  |  |  |  |  |  |  |  |
| Cao et al. (2012) |  |  | Hippo | Hippo |  |  |  |  |  |  |  |  |  |  |
| Chen et al. (2021) |  |  |  |  |  |  |  |  |  |  |  |  | FFG |  |
| Chen et al. (2020) |  | Amyg |  |  |  |  |  |  |  |  |  |  |  |  |
| Chen et al. (2015) |  |  | PCC | IPL |  |  |  |  |  |  |  |  |  |  |
| Crowther et al. (2015) |  |  | PCUN |  |  |  |  | Ins DACC |  | IPS |  | DLPFC |  |  |
| Cui et al. (2020) |  |  |  | SFGmed ACC |  |  |  |  |  |  |  | OFC |  |  |
| Davey et al. (2012) |  |  | ACC | ACC |  |  |  |  |  |  |  |  |  |  |
| de Kwaasteniet et al. (2013) |  |  | ACC | ACC |  |  |  |  |  |  |  |  |  |  |
| de Kwaasteniet et al. (2015) |  |  |  | SFGmed |  |  |  |  |  |  |  | DLPFC |  |  |
| Duan et al. (2020) | Amyg | Amyg |  |  |  |  |  |  |  |  |  |  |  |  |
| Fettes et al. (2018) |  |  | LFPC | MFC LFPC |  |  |  |  |  |  |  |  |  |  |
| Frodl et al. (2010) |  |  |  |  |  |  |  |  |  |  |  | OFC |  |  |
| Furman et al. (2011) |  | STR | CAU | CAU |  |  |  | PUT |  |  | PUT | PUT |  |  |
| Ge et al. (2019) |  |  |  | Hippo |  |  |  |  |  |  |  |  |  |  |
| Guo et al. (2015a) |  |  | Cereb |  |  |  |  |  |  |  |  |  |  |  |
| Guo et al. (2015b) |  |  |  |  |  |  |  | Ins |  |  |  |  |  |  |
| Hao et al. (2020) |  |  |  | Hippo |  |  |  |  |  |  |  |  |  |  |
| He et al. (2019) |  |  | Hippo | ACC CAU Hippo |  |  |  |  |  |  |  | VLPFC PUT |  |  |
| Hu et al. (2019) |  |  |  |  |  |  |  |  |  |  |  |  | Ins | Ins |
| Hu et al. (2023) |  | NACC |  |  |  |  |  |  |  |  |  |  |  |  |
| Krug et al. (2022) |  |  | Hippo |  |  |  |  | Ins |  |  |  | IPL |  |  |
| Lee et al. (2019) | Parahip |  |  |  |  |  |  |  |  | DAN |  |  |  |  |
| Li et al. (2021) |  |  | PCC Hippo | PCC |  |  |  |  |  |  |  |  |  |  |
| Lin et al. (2022) |  |  |  | ACC |  |  |  |  |  |  |  |  |  |  |
| Liu et al. (2012) |  |  | Cereb | Cereb |  |  |  | Cereb |  |  | Cereb | Cereb |  |  |
| Liu et al. (2020) |  | Amyg | Hippo | Hippo PCC Cereb ITG |  |  |  |  |  |  |  | APFC |  |  |
| Long et al. (2020) |  |  |  |  | LG | LG |  |  |  |  |  |  |  |  |
| Peng et al. (2015) |  |  |  | PCUN |  |  |  |  |  |  |  |  |  |  |
| Penner et al. (2018) |  |  |  |  |  |  | TPJ | TPJ | TPJ | TPJ |  |  | TPJ |  |
| Ramasubbu et al. (2014) | Amyg | Amyg |  |  |  |  |  |  |  |  |  |  |  |  |
| Sawaya et al. (2015) |  |  | OPFC | SFGmed |  |  |  |  |  |  |  |  |  |  |
| Tang et al. (2019) | Amyg | Amyg |  |  |  |  |  |  |  |  |  |  |  |  |
| Tang et al. (2013) |  | Amyg |  |  |  |  |  |  |  |  |  |  |  |  |
| Todeva-Radneva et al. (2023) |  |  |  |  |  |  |  | ACC |  |  |  |  |  |  |
| Wang et al. (2019) |  | Hypo |  |  |  |  |  |  |  |  |  |  |  |  |
| Wang et al. (2020) |  |  |  |  |  |  |  |  |  |  |  | DLPFC |  |  |
| Wang et al. (2016) |  | Amyg |  |  |  |  |  |  |  |  | DLPFC | DLPFC |  |  |
| Wu et al. (2016) |  |  |  |  |  |  |  | DACC |  |  |  |  |  |  |
| Wu et al. (2022) |  |  | ACC | ACC |  |  |  | ACC |  |  | ACC | ACC |  |  |
| Yang et al. (2017) |  |  | CAU | CAU |  |  |  |  |  |  |  |  |  | STG |
| Yang et al. (2016) |  |  |  |  |  |  |  |  |  |  | Ins | Ins |  |  |
| Ye et al. (2012) |  |  |  |  |  |  |  |  |  |  | DLPFC | DLPFC |  |  |
| Zhang et al. (2022a) |  | ACC | ACC | ACC |  |  |  |  |  |  |  |  |  |  |
| Zhang et al. (2022b) |  | NACC |  |  |  |  |  |  |  |  | CAU | CAU |  |  |
| Zhang et al. (2022c) |  |  |  |  |  |  |  |  |  |  | DLPFC | DLPFC |  |  |
| Zhou et al. (2010) |  |  | DLFG MTG PCC SFGmed LPL | DLFG LOFC MTG Cereb SFGmed LPL STG Parahip |  |  |  | Ins |  |  | IFG | IFG IPL |  |  |
| Zhou et al. (2022) | NACC | NACC |  |  |  |  |  |  |  |  |  |  |  |  |
| Zhu et al. (2018) |  |  |  | PCUN |  |  |  |  |  |  |  |  |  |  |
| Zu et al. (2019) |  | Amyg |  |  |  |  |  |  |  |  |  |  |  |  |
| Total | 5 | 14 | 20 | 24 | 1 | 1 | 2 | 11 | 1 | 4 | 9 | 18 | 3 | 2 |

Note: “B” represents the between-network group, indicating functional connectivity between different networks; “W” represents the within-network group, indicating functional connectivity within the same network.

Abbreviations: ACC, anterior cingulate cortex; Amyg, amygdala; APFC, anterior prefrontal gyrus; CAU, caudate nucleus; Cereb, cerebellum; DACC, dorsal anterior cingulate cortex; DAN, dorsal attention network; DLFC, dorsolateral frontal cortex; DLPFC, dorsolateral prefrontal cortex; DMPFC, dorsomedial prefrontal cortex; DMN, default mode network; FFG, fusiform gyrus; FPN, frontoparietal network; Hippo, hippocampus; IFG, inferior frontal gyrus; IPL, inferior parietal cortex; ITG, inferior temporal gyrus; Ins, insula; LFG, lateral frontal gyrus; LG, lingual gyrus; LN, limbic network; LOFC, lateral orbitofrontal cortex; LPL, lateral parietal lobule; MFC, medial frontal polar cortex; MTG, middle temporal gyrus; NACC, nucleus accumbens; OFC, orbitofrontal cortex; OPFC, orbital prefrontal cortex; Parahip, parahippocampal; PCC, posterior cingulate cortex; PCUN, precuneus; PUT, putamen; SFGmed, medial superior frontal gyrus; SMN, somatosensory network; STG, superior temporal gyrus; STR, striatum; TPJ, temporoparietal junction; VAN, ventral attention network; VN, visual network; VLPFC, ventrolateral prefrontal cortex.

Table S7. Summary of information included in ICA studies in this meta-analysis.

| Studies | Resting-state networks | | | | | | |
| --- | --- | --- | --- | --- | --- | --- | --- |
|  | LN | DMN | VN | VAN | DAN | FPN | SMN |
| Dong et al. (2019) | No | Yes | No | No | No | Yes | No |
| Lu et al. (2020) | No | No | Yes | No | No | No | Yes |
| Luo et al. (2021) | No | No | Yes | No | Yes | Yes | No |
| Shi et al. (2015) | No | Yes | No | Yes | No | Yes | No |
| Yu et al. (2021) | No | Yes | No | No | No | No | No |
| Zhu et al. (2012) | No | Yes | Yes | No | No | Yes | No |
| Total | 0 | 4 | 3 | 1 | 1 | 4 | 1 |

Note: “Yes” indicates that the article studied this network, while “No” indicates that the study did not explore this network.

Abbreviations: DAN, dorsal attention network; DMN, default mode network; FPN, frontoparietal network; ICA, independent component analysis; LN, limbic network; SMN, somatosensory network; VAN, ventral attention network; VN, visual network.

Table S8. Meta-regression analysis: associations of RSN alterations with illness duration and HAMD scores in depression.

| Seed-network | Target-network | Target-anatomy | Peak MNI coordinates | | | Meta regression *p*-value | |
| --- | --- | --- | --- | --- | --- | --- | --- |
|  |  |  | X | Y | Z | Duration | HAMD |
| Within-network | | | | | | | |
| DMN | DMN | L-PCU | -4 | -68 | 44 | 0.6422 | 0.0561 |
| DMN | DMN | R-ANG/R-MTG | 48 | -60 | 28 | 0.0805 | 0.4924 |
| DMN | DMN | L-ANG | -38 | -72 | 38 | 0.7298 | 0.7784 |
| FPN | FPN | L-CAU | -6 | -2 | 10 | 0.7997 | 0.7509 |
| Between-network | | | | | | | |
| DMN | FPN | R-MFG/R-IFG | 44 | 34 | 24 | 0.7613 | 0.3809 |
| DMN | SMN | R-STG/R-HES | 44 | -16 | 8 | 0.9574 | 0.5157 |
| LN | DMN | L-MTG/L-ITG | -46 | -2 | -24 | 0.0547 | 0.1704 |
| LN | FPN | L-CAU | -14 | 4 | 22 | 0.2430 | 0.1457 |
| VAN | DAN | R-IPL/R-SPL | 30 | -50 | 54 | **0.0013** | 0.9878 |
| VAN | DAN | R-MOG | 46 | -74 | 28 | 0.2230 | 0.0602 |

Note: Values in bold and underlined indicate statistically significant associations (*p* < 0.05).

Abbreviations: DAN, dorsal attention network; DMN, default mode network; FPN, frontoparietal network; HAMD, Hamilton Depression Rating Scale; L-ANG, left angular gyrus; L-CAU, left caudate nucleus; L-ITG, left inferior temporal gyrus; L-MTG, left middle temporal gyrus; L-PCU, left precuneus; LN, limbic network; MNI, Montreal Neurological Institute; R-ANG, right angular gyrus; R-HES, right Heschl’s gyrus; R-IFG, right inferior frontal gyrus; R-IPL, right inferior parietal lobule; R-MFG, right middle frontal gyrus; R-MOG, right middle occipital gyrus; R-MTG, right middle temporal gyrus; R-SPL, right superior parietal lobule; R-STG, right superior temporal gyrus; RSN, resting-state network; SMN, somatosensory network; VAN, ventral attention network.

Supplementary Figures

**
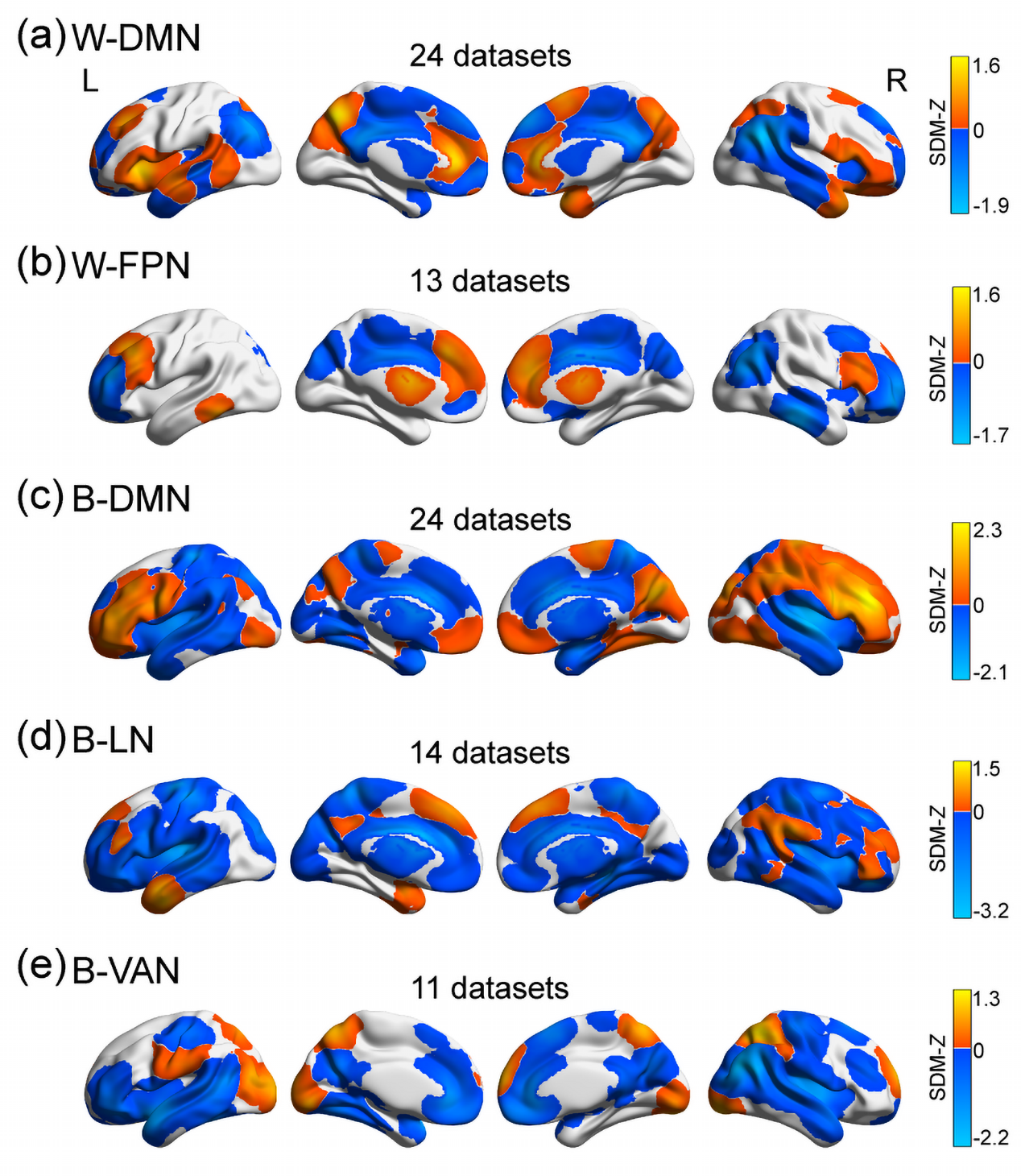
**

Figure S1. Meta-analysis results of significant RSN changes in depression (unthresholded).

The results are presented as follows: (a) within the DMN, (b) within the FPN, (c) between the DMN and other networks, (d) between the LN and other networks, and (e) between the VAN and other networks. The colorbar represents SDM-*Z* values, with warm and cold colors indicating increased and decreased functional connectivity in patients with depression, respectively.

Abbreviations: B, between; DMN, default mode network; FPN, frontoparietal network; LN, limbic network; SDM, seed-based *d* mapping; VAN, ventral attention network; W, within.


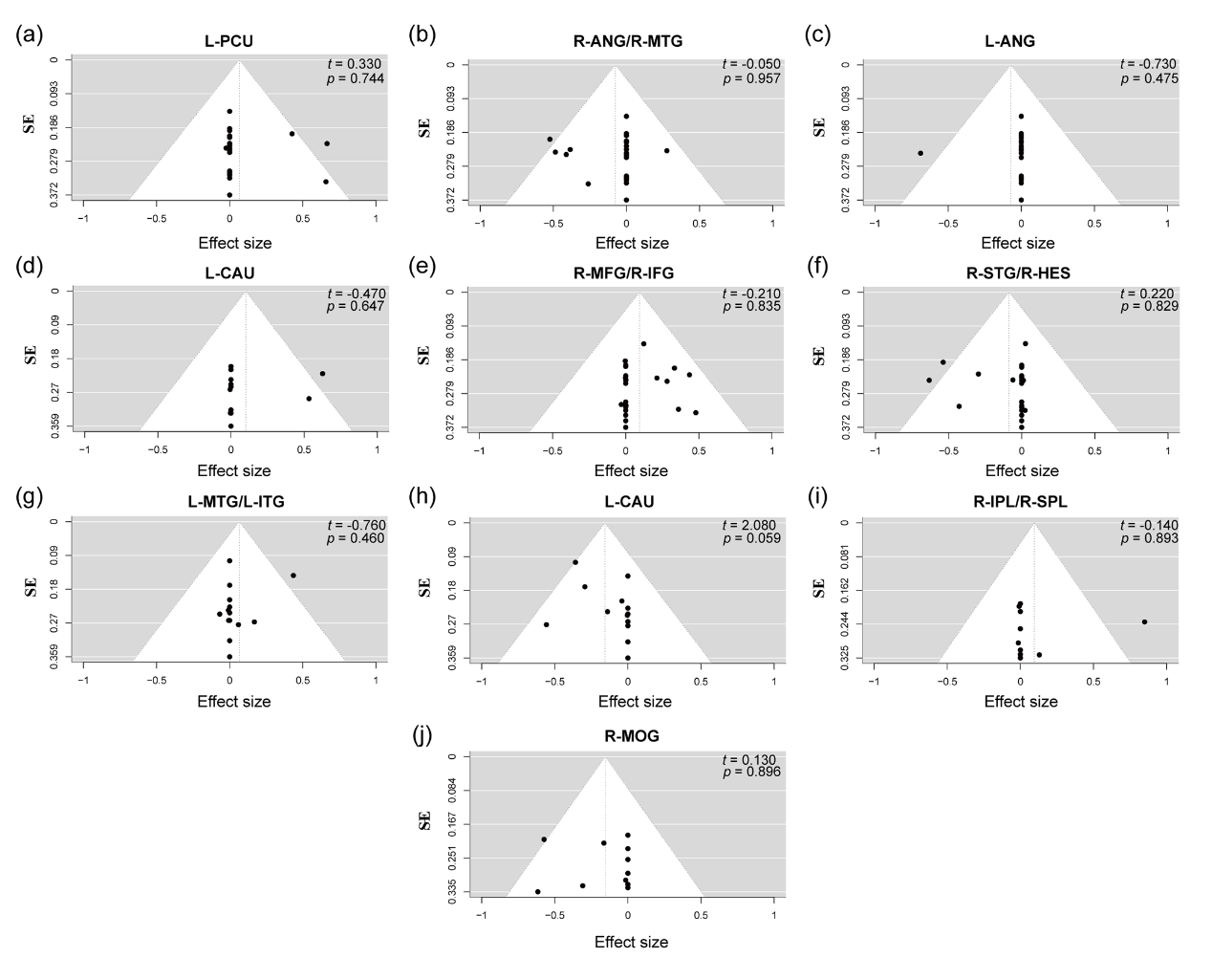


Figure S2. Funnel plots of regions with significantly altered functional connectivity.

The funnel plots illustrate the relationship between effect size and standard error, with each study represented by a point. Specifically, the results are grouped as follows: within the DMN include (a) L-PCU, (b) R-ANG/R-MTG, and (c) L-ANG; within the FPN include (d) L-CAU; between the DMN and other networks include (e) R-MFG/R-IFG and (f) R-STG/R-HES; between the LN and other networks include (g) L-MTG/L-ITG and (h) L-CAU; and between the VAN and other networks include (i) R-IPL/R-SPL and (j) R-MOG. These plots provide an intuitive overview of the variability in effect sizes across studies for each brain region.

Abbreviations: DMN, default mode network; FPN, frontoparietal network; L-ANG, left angular gyrus; L-CAU, left caudate nucleus; L-ITG, left inferior temporal gyrus; L-MTG, left middle temporal gyrus; LN, limbic network; L-PCU, left precuneus; R-ANG, right angular gyrus; R-HES, right Heschl’s gyrus; R-IFG, right inferior frontal gyrus; R-IPL, right inferior parietal lobule; R-MFG, right middle frontal gyrus; R-MOG, right middle occipital gyrus; R-MTG, right middle temporal gyrus; R-SPL, right superior parietal lobule; R-STG, right superior temporal gyrus; SE, standard error; VAN, ventral attention network.


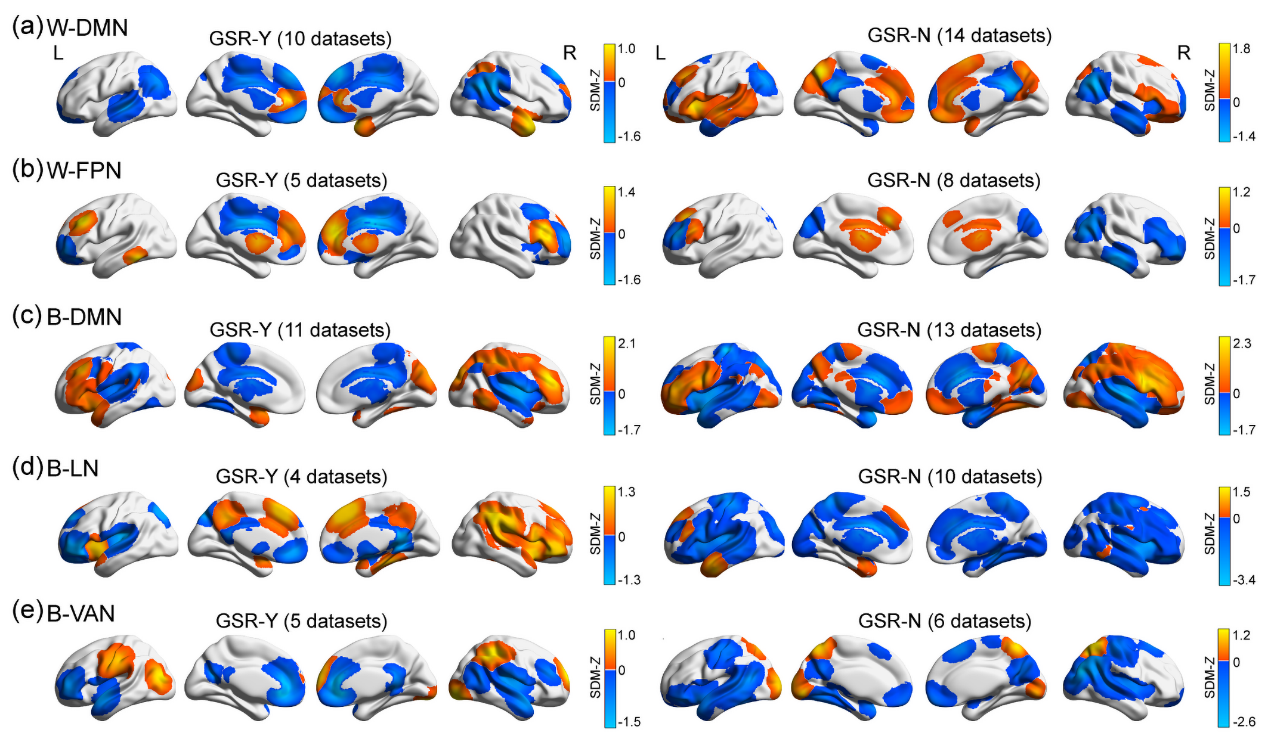


Figure S3. Subgroup analysis of the effect of GSR on functional connectivity in depression.

The figure presents the results of subgroup meta-analyses examining the differences in functional connectivity findings in patients with depression based on the application of GSR (GSR-Y: applied, GSR-N: not applied). The results are as follows: (a) within-DMN, (b) within-FPN, (c) between DMN and other networks, (d) between LN and other networks, and (e) between VAN and other networks. The colorbar represents SDM-*Z* values, with warm colors indicating increased functional connectivity and cold colors indicating decreased functional connectivity. Given the limited number of studies and the exploratory nature of the analysis, the results are uncorrected and unthresholded to avoid overinterpretation and provide a more comprehensive data visualization.

Abbreviations: B, between; DMN, default mode network; FPN, frontoparietal network; GSR, global signal regression; LN, limbic network; SDM, seed-based *d* mapping; VAN, ventral attention network; W, within.


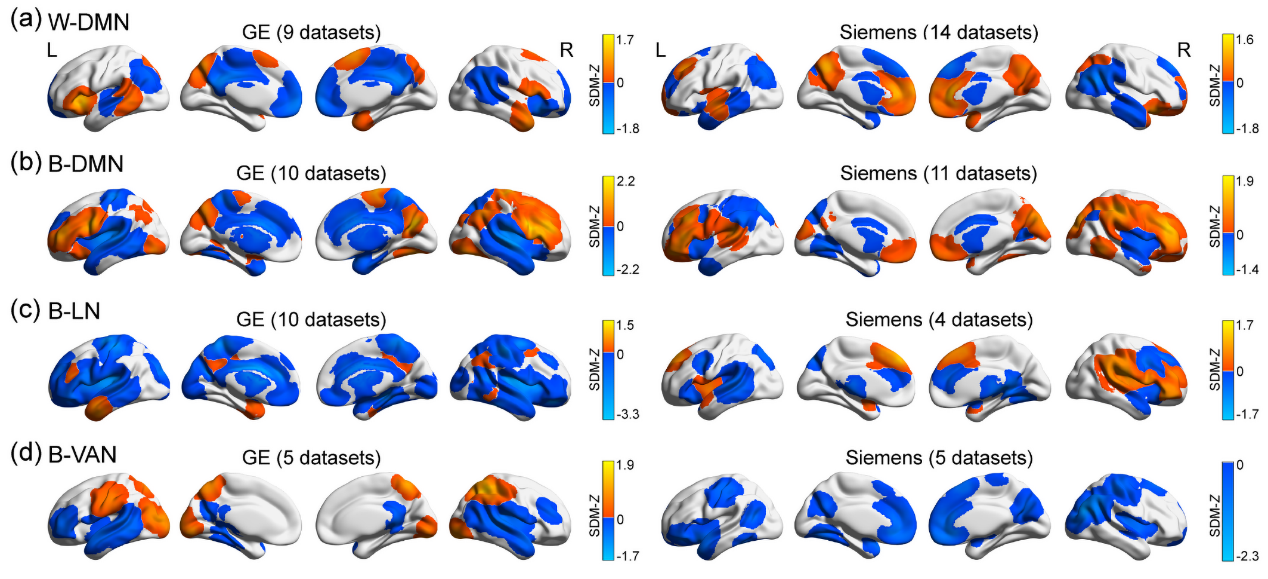


Figure S4. Subgroup analysis of the impact of MRI scanner type (GE vs. Siemens) on functional connectivity in depression.

The figure presents the results of subgroup meta-analyses examining the differences in functional connectivity findings in patients with depression based on scanner manufacturer (GE vs. Siemens, Philips scanners were excluded due to insufficient data for analysis). Due to all 13 studies of W-FPN being conducted on Siemens scanners, the analyses focus on the remaining four groups: (a) within-DMN, (b) between DMN and other networks, (c) between LN and other networks, and (d) between VAN and other networks. The colorbar represents SDM-*Z* values, with warm colors indicating increased functional connectivity and cold colors indicating decreased functional connectivity. Given the limited number of studies and the exploratory nature of the analysis, the results are uncorrected and unthresholded to avoid overinterpretation and provide a more comprehensive data visualization.

Abbreviations: B, between; DMN, default mode network; LN, limbic network; SDM, seed-based *d* mapping; VAN, ventral attention network; W, within.

References for Supplementary Materials

Lin, J., Li, L., Pan, N., Liu, X., Zhang, X., Suo, X., . . . Gong, Q. (2023). Neural correlates of neuroticism: A coordinate-based meta-analysis of resting-state functional brain imaging studies. *Neurosci Biobehav Rev, 146*, 105055. doi:10.1016/j.neubiorev.2023.105055

Norman, L. J., Carlisi, C., Lukito, S., Hart, H., Mataix-Cols, D., Radua, J., & Rubia, K. (2016). Structural and Functional Brain Abnormalities in Attention-Deficit/Hyperactivity Disorder and Obsessive-Compulsive Disorder: A Comparative Meta-analysis. *JAMA Psychiatry, 73*(8), 815-825. doi:10.1001/jamapsychiatry.2016.0700

Shepherd, A. M., Laurens, K. R., Matheson, S. L., Carr, V. J., & Green, M. J. (2012). Systematic meta-review and quality assessment of the structural brain alterations in schizophrenia. *Neurosci Biobehav Rev, 36*(4), 1342-1356. doi:10.1016/j.neubiorev.2011.12.015
